# Three-Dimensional Zirconia-Based Scaffolds for Load-Bearing Bone-Regeneration Applications: Prospects and Challenges

**DOI:** 10.3390/ma14123207

**Published:** 2021-06-10

**Authors:** Kumaresan Sakthiabirami, Vaiyapuri Soundharrajan, Jin-Ho Kang, Yunzhi Peter Yang, Sang-Won Park

**Affiliations:** 1 Department of Prosthodontics, Dental Science Research Institute, School of Dentistry, Chonnam National University, Gwangju 61186, Korea; sakthikarthi.dentist@gmail.com (K.S.); jhk.bme1002@gmail.com (J.-H.K.); 2 Department of Materials Science and Engineering, Chonnam National University, Gwangju 61186, Korea; soundharajan.007@gmail.com; 3 Department of Orthopaedics Surgery, Stanford University, Stanford, CA 94305-5110, USA; ypyang@stanford.edu

**Keywords:** 3D zirconia-based scaffold, bone-regeneration applications, composite, coating, fabrication techniques, bioceramics

## Abstract

The design of zirconia-based scaffolds using conventional techniques for bone-regeneration applications has been studied extensively. Similar to dental applications, the use of three-dimensional (3D) zirconia-based ceramics for bone tissue engineering (BTE) has recently attracted considerable attention because of their high mechanical strength and biocompatibility. However, techniques to fabricate zirconia-based scaffolds for bone regeneration are in a stage of infancy. Hence, the biological activities of zirconia-based ceramics for bone-regeneration applications have not been fully investigated, in contrast to the well-established calcium phosphate-based ceramics for bone-regeneration applications. This paper outlines recent research developments and challenges concerning numerous three-dimensional (3D) zirconia-based scaffolds and reviews the associated fundamental fabrication techniques, key 3D fabrication developments and practical encounters to identify the optimal 3D fabrication technique for obtaining 3D zirconia-based scaffolds suitable for real-world applications. This review mainly summarized the articles that focused on in vitro and in vivo studies along with the fundamental mechanical characterizations on the 3D zirconia-based scaffolds.

## 1. Introduction

### 1.1. General Overview of Zirconia Bioceramics

Zirconium dioxide (zirconia) was first discovered by the German chemist Martin Heinrich Klaproth in 1789 [1]. The use of zirconia in the biomedical field emerged in 1969, as it is a promising alternative to alumina and metal for the construction of hip prosthesis in orthopaedic applications [2]. Zirconia is an oxide form of zirconium (strong transition metal), which does not exist in pure form in the Earth’s crust. It is found in the minerals baddeleyite and zircon. Zirconia is a polycrystalline ceramic that exhibits three different crystallographic phases depending on the temperature and pressure: monoclinic (M), tetragonal (T) and cubic (C) [3]. Pure zirconia with a monoclinic structure is stable at room temperature and up to 1170 °C and has inferior mechanical properties to the other two phases. The transition from the monoclinic phase to the tetragonal phase occurs between 1170 °C and 2370 °C, accompanied by approximately 4–5 vol% reduction. Zirconia shrinks to the cubic phase above 2370 °C and up to the melting point (2680 °C) [4]. The transformation of the tetragonal zirconia lattice into the monoclinic phase occurs with approximately 3–4% volume expansion upon cooling. This phase-transformation behaviour of zirconia results in crack propagation over time, because of the internal stress produced during cooling. The aforementioned phenomena can be inhibited by the addition of a relative amount of metallic oxides (also known as ‘dopants’ or ‘stabilising oxides’) such as Y_2_O_3_, MgO, CaO and CeO_2_ [5]. This doped zirconia exhibits a unique property known as ‘transformation toughening’, and it is considered a key advantage for biomedical applications in orthopaedics and dentistry [5,6].

### 1.2. Inevitability of Widespread Use of Zirconia Bioceramics in Biomedical Applications

It is well known that zirconia is available in various chemical forms; however, in the field of biomedical research, only three types have primarily been used: yttrium-stabilised tetragonal zirconia polycrystals (3Y-TZP), magnesium-doped partially stabilised zirconia (Mg-PSZ) and zirconia-toughened alumina (ZTA) are the major contributors in biomedical and dental applications [7]. The use of zirconia bioceramics in dental applications in the form of dental prostheses started in the early 1980s and has attracted considerable attention in the dental community [5,8]. In the early days, wide variety metal alloys were employed in dental applications; titanium alloys exhibited clinical success rates of 92–98% yet had minor shortcomings corrosion induced metal ion dissolution into body fluids. [9,10]. The arrival of zirconia bioceramics was a blessing to dentistry owing to their tooth-like colour, high fracture toughness and low-temperature conductance. Owing to its high flexural strength (900 MPa) and non-reactivity with body fluids, researchers successfully employed zirconia for crown and bridge applications [11,12]. Extensive research attempts have been made to utilise the mechanical and biological advantages of zirconia in the form of dental posts as a synthetic tooth root to replace missing teeth [13,14]. Numerous reviews of the use of zirconia for implant applications have been conducted and served as a potential reference for the dental and orthopedic research community [3,7,9,10].

More importantly, after the effectual utilisation of zirconia ceramics for the construction of tooth reinforced repairs, clinicians extended the use of the valuable features of zirconia ceramics (that is, the lower elastic modulus and higher toughness for implant-reinforced renovations) [15]. After the commercialisation of zirconia dental implants in 1987 by Sigma Implants (Sandhause, Incermed, Lausanne, Switzerland), zirconia implants became widely accessible. However, the use of zirconia scaffolds for comprehensive load-bearing applications is commercially nonviable owing to various challenges, such as scaffold fabrication and surface modification. The promising development of zirconia implants has been documented worldwide, and several reviews have been conducted in recent years. For instance, Soon et al., and Yin et al., summarised the recent advances in fabrication techniques for zirconia implants [16,17]. Cionca et al., and Amleh et al., compared the clinical advantages and difficulties between zirconia and titanium implants in the previous review [18,19]. These reviews provide a basic understanding of the origin and evolution of zirconia bioceramics from dental prostheses to biomedical implant applications.

### 1.3. Commencement of Zirconia over Calcium-Phosphate Scaffolds in Bone-Regeneration Applications

Bone-tissue regeneration using synthetic biomaterials with an identical chemical composition to human bone has developed as a pioneering and favourable strategy for the restoration of bone defects [20]. Calcium phosphate (CP) has chemical and biological behaviours identical to those of natural bone, and calcium phosphate-based materials have been extensively studied by various research groups worldwide [21,22,23]. However, they have their own faintness in terms of mechanical properties, which is a major concern for load-bearing applications [24]. More importantly, CP-based materials are biodegradable in nature when subjected to the human body and fail to support the reconstruction process because they do not maintain their original shapes [25]. To overcome this calamity, researchers used a composite formation strategy, blending calcium phosphate with mechanically strong and biologically inert zirconia [26]. It is well known that zirconia has an elastic modulus, fracture toughness and osseointegration properties similar to those of human bones [27]. Since traditional two-dimensional (2D) biomaterials cannot retain three-dimensional (3D) architectures, 2D designs have a limited ability to mimic the multidimensional extracellular background, which is essential for promoting cell feasibility and functionality [28]. The design of synthetic bone-graft materials in the form of 3D porous scaffolds loaded with tissue-activating features or precise cells to launch bone restoration is an innovative approach [29,30].

In recent times, considerable research attention has been focused on the progress of engineering techniques for developing CP biomaterials in the form of 3D scaffolds [31,32]. In a recent review, Ryan et al., reported that the available 3D-printing techniques have matured sufficiently to formulate 3D porous CP scaffolds [33]. Additionally, they stated that 3D-printed CP scaffolds must reach the level 1 preclinical stage to confirm effectiveness in a large animal before bone-regeneration checks are performed for human trials. However, the synthesis of 3D zirconia-based scaffolds remains in a stage of infancy because of the lack of availability of 3D fabrication techniques [34].

Few 3D fabrication techniques have been successfully investigated for fabricating 3D zirconia-based scaffolds.

**Scheme 1:**
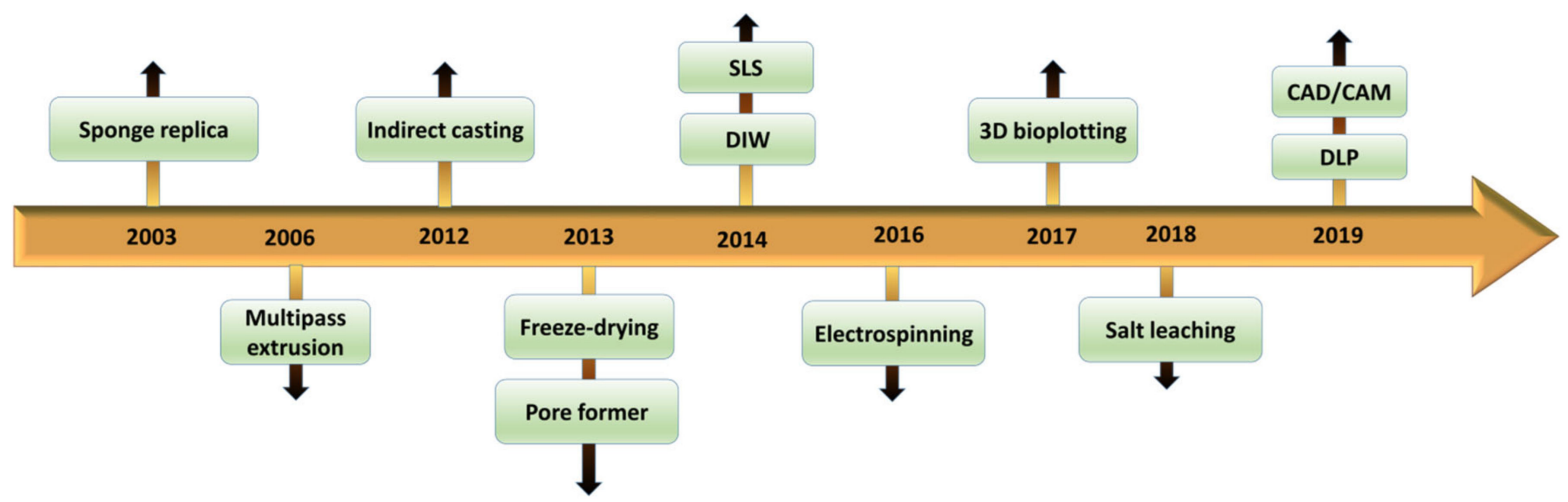
Evolution of 3D zirconia-based scaffolds (with regard to the scaffold fabrication technique) for bone-regeneration applications.

**Scheme 2:**
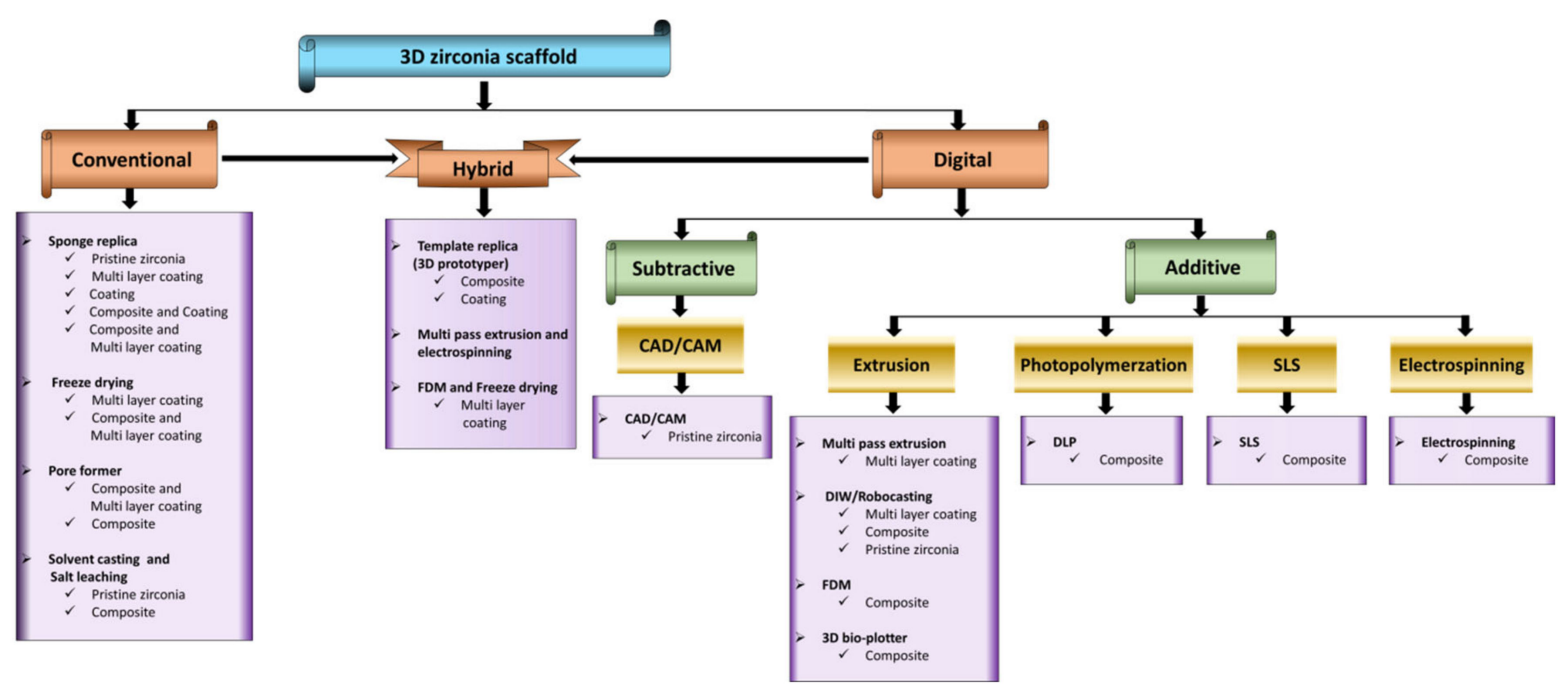
Overview of the 3D fabrication techniques for zirconia-based scaffolds, along with the surface optimisation tactics using coating and composite strategies.

The evolution of the fabrication techniques for the fabrication of 3D zirconia-based scaffolds over time is presented in Scheme 1. As shown, 3D zirconia-based scaffold fabrication via the sponge replica technique for bone-regeneration application started in the late 2000s. However, the use of modern 3D fabrication techniques for 3D zirconia-based scaffolds started very recently. According to the types of 3D fabrication techniques, this article can be divided into three main sections: conventional, hybrid and digital (Scheme 2).

**Scheme 3:**
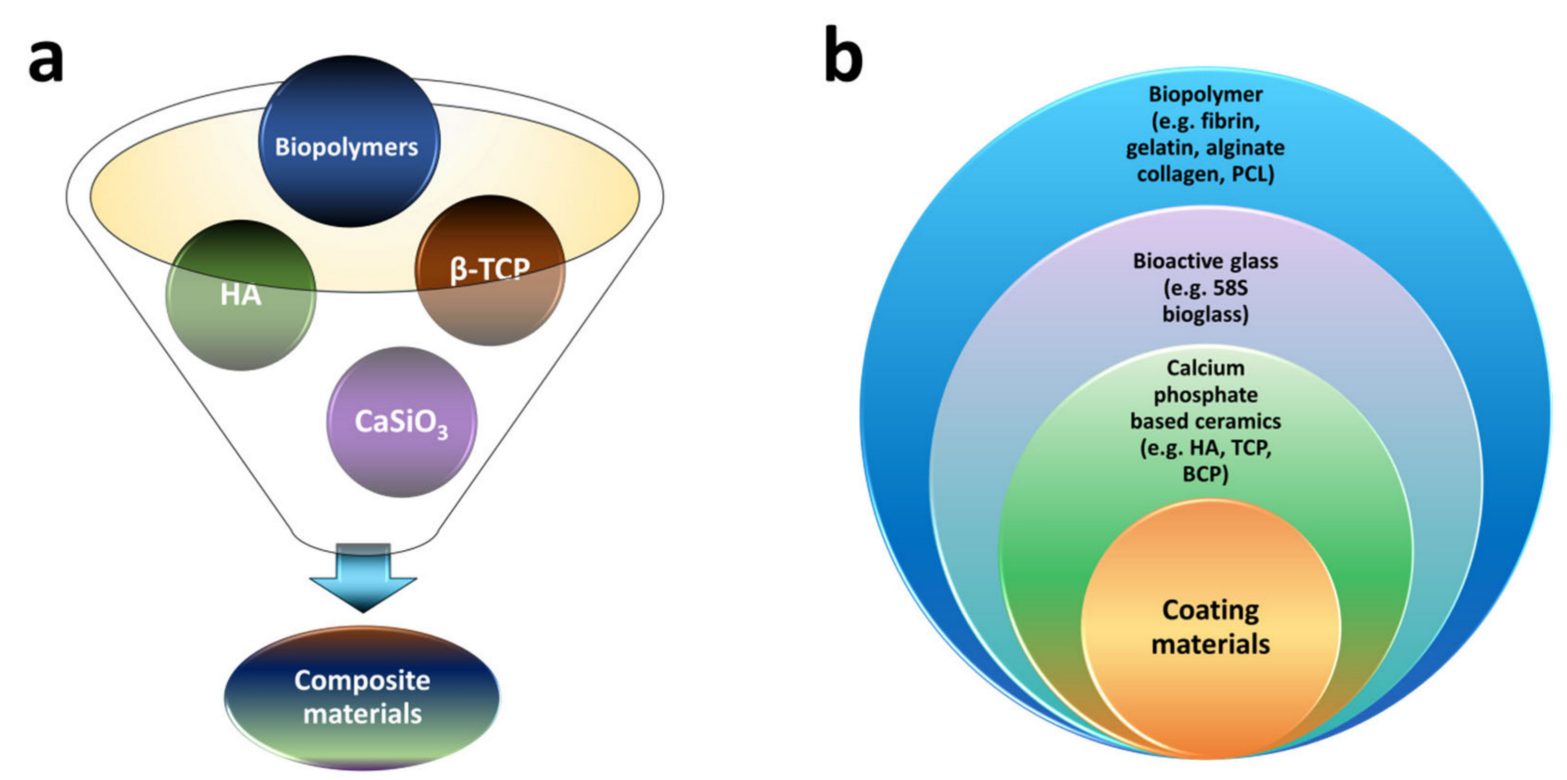
(**a**) Composite materials and (**b**) coating materials for bioengineered zirconia-based scaffolds.

Originally, researchers investigated zirconia-based scaffolds using conventional moulding approaches, including sponge replicas, freeze-drying, pore-formers and salt leaching. Even though the conventional techniques for fabricating zirconia-based scaffolds are economical, the precise formation of complex structures is difficult [34]. Thus, in recent years, researchers have used 3D digital techniques as alternative methods for fabricating zirconia-based scaffolds, as for other biomaterials. In the following sections, we discuss the roles of previously reported composite/coating approaches in enhancing the mechanical and biological properties of zirconia-based scaffolds. The most common composite and coating materials used for the bioengineering of zirconia-based scaffolds are presented in Scheme 3.

Thus, this review systematically summarises the challenges and advancements in the development of 3D fabrication techniques for 3D zirconia scaffolds and their role in bone-regeneration applications for the first time as per the author’s knowledge. We focused on studies in which the biological activity of zirconia scaffolds was successfully demonstrated in vitro and in vivo. Finally, guidance for future research directions for formulating optimal zirconia scaffolds via conventional and modern 3D digital techniques is provided. More importantly, we comprehensively addressed the gap between the academic reliability and clinical reliability of various 3D zirconia scaffold techniques for bone regeneration. We hope that this review will draw attention to the production of 3D zirconia scaffolds and promote their clinical implementation.

## 2. 3D Zirconia-Based Scaffolds via Conventional Technique

### 2.1. Sponge Replica Technique

The polymeric sponge replica method is the most popular technique for producing interconnected porous bioceramics. This method is known for its simplicity. It involves the coating of open-cell polymeric foam with the desired bioceramic slurry and subsequent thermal treatment to burn-out the polymeric foam and obtains a bioceramic with a 3D structure similar to that of the original polymeric foam [35]. The composite/coating design mixture of biocompatible materials and bioinert porous zirconia-based scaffolds have been studied extensively by multiple research teams [36]. By fixing bioinert zirconia scaffolds as a mainstream loadbearing framework, several scholars have fabricated advanced coating/composite formations using biocompatible and osteoinductive bioceramics.

Hydroxyapatite (Ca_10_(PO_4_)_6_(OH_2_), HA) is a well-known material in the calcium-phosphate family owing to its analogous properties to human bone components and has been employed for bone replacement [37]. Tricalcium phosphate (β-TCP), which is commonly referred to as bone ash, has a chemical formula of Ca_3_(PO_4_)_2_. Similar to HA, TCP is rich in calcium and phosphorus, which can induce new-bone construction [38]. β-TCP has been formulated directly in the form of scaffolds and tested for bone-tissue regeneration applications [39]. Similarly, the biphasic calcium phosphate (BCP) bioceramic—a mixture of TCP (Ca_3_(PO_4_)_2_) and HA (Ca_10_(PO_4_)_6_(OH_2_)—has been used as a bone-craft material because it has a good chemical bone-bonding property and a greater bioresorption capability than HA and TCP individually [40]. BCP promotes the growth of osteoblasts/osteoclasts in a more habitual fashion than pristine HA and TCP [41]. Our literature assessment indicated that HA, TCP and BCP are the calcium-phosphate ceramics that are predominantly employed to formulate bioengineered zirconia scaffolds. In the following sections, we comprehensively discuss the approaches and scientific advancements of calcium-phosphate incorporation into zirconia scaffolds and zirconia incorporation into calcium-phosphate scaffolds, which have been investigated by numerous bone-tissue engineers.

Initiation of the application of CP-altered zirconia-based scaffolds for bioactivity, an enhancement was started in 2003. Kim et al., proposed a biocompatible HA coating (dip-coating) on the surface of a zirconia scaffold (synthesised via the sponge replication technique) [42]. In particular, to minimise the chemical conversion reaction between HA and zirconia, a fluorapatite (FA) layer was adopted as an intermediate. The authors found that the strength of the HA-coated zirconia scaffolds increased by a factor of 7 (compared with the HA), which is encouraging for load-bearing applications. Furthermore, the in vitro results confirmed that the surface-treated porous zirconia scaffolds significantly promoted the growth and proliferation of the osteoblast cells compared with untreated zirconia scaffolds. It is known that FA-inserted HA surface-treated zirconia scaffolds provide enriched bone-tissue regeneration properties. In 2004, Kim et al., varied the external coating with different calcium-phosphate coatings via the powder slurry method (TCP, HA, FA) or in the form of composites including TCP + HA and HA + FA on sponge replication technique-derived zirconia scaffolds [43]. Before the direct CP coating was formed, the zirconia scaffolds were coated with FA as an intermediate layer to minimise the interaction between the CP and the zirconia scaffolds. The authors found that the cell evolutions depended on the coating environment. For example, from the cell-differentiation output of MG63 cells, the alkaline phosphate establishment was found to be enriched in surface-treated scaffolds HA (HA, HA + FA and HA + TCP) compared with pure TCP- and FA-coated scaffolds. In 2004, the same group studied the effects of coating zirconia with HA via the sol-gel and slurry methods [44]. The dissolution rate in the case of the sol-gel-slurry coating was higher than that for the slurry coating. Additionally, the authors controlled the dissolution rate by adjusting the annealing temperature of the sol-gel HA layer. Osteoblast proliferation was confirmed by biological assessments using human osteoblast-like cells (MG63 cells).

After the successful formulation of zirconia scaffolds into strong and bioactive scaffolds via apatite dual-layers inner FA layer and an HA outer layer on the zirconia scaffolds, Kim et al., further authorised the clinical prospects of bioengineered zirconia scaffolds by performing in vivo studies on a rabbit calvarial defect model in 2008 [45]. To recognize the geometrical impacts on the bone-regeneration activities, the authors varied the porosity and pore size of the scaffolds. Remarkably, the bioengineered scaffold exhibited a higher porosity (~84–87%) and compressive strength (~7–8 MPa) than pure apatite-based scaffolds (~74% and ~2 MPa, respectively). Furthermore, according to the in vivo studies on the rabbit calvarial defect model, they proposed that the new-bone construction ensued effectively within the pore channels of all the apatite-engineered zirconia scaffolds, where the bone regeneration phases were similar to those of the pristine HA scaffold. Thus, Kim et al., confirmed the capability of apatite-engineered zirconia scaffolds for bone-regeneration applications by succeeding fundamental studies from in vitro to in vivo amendments, which is crucial for realising any academic research activities persevering further to real-world standards.

**Figure 1:**
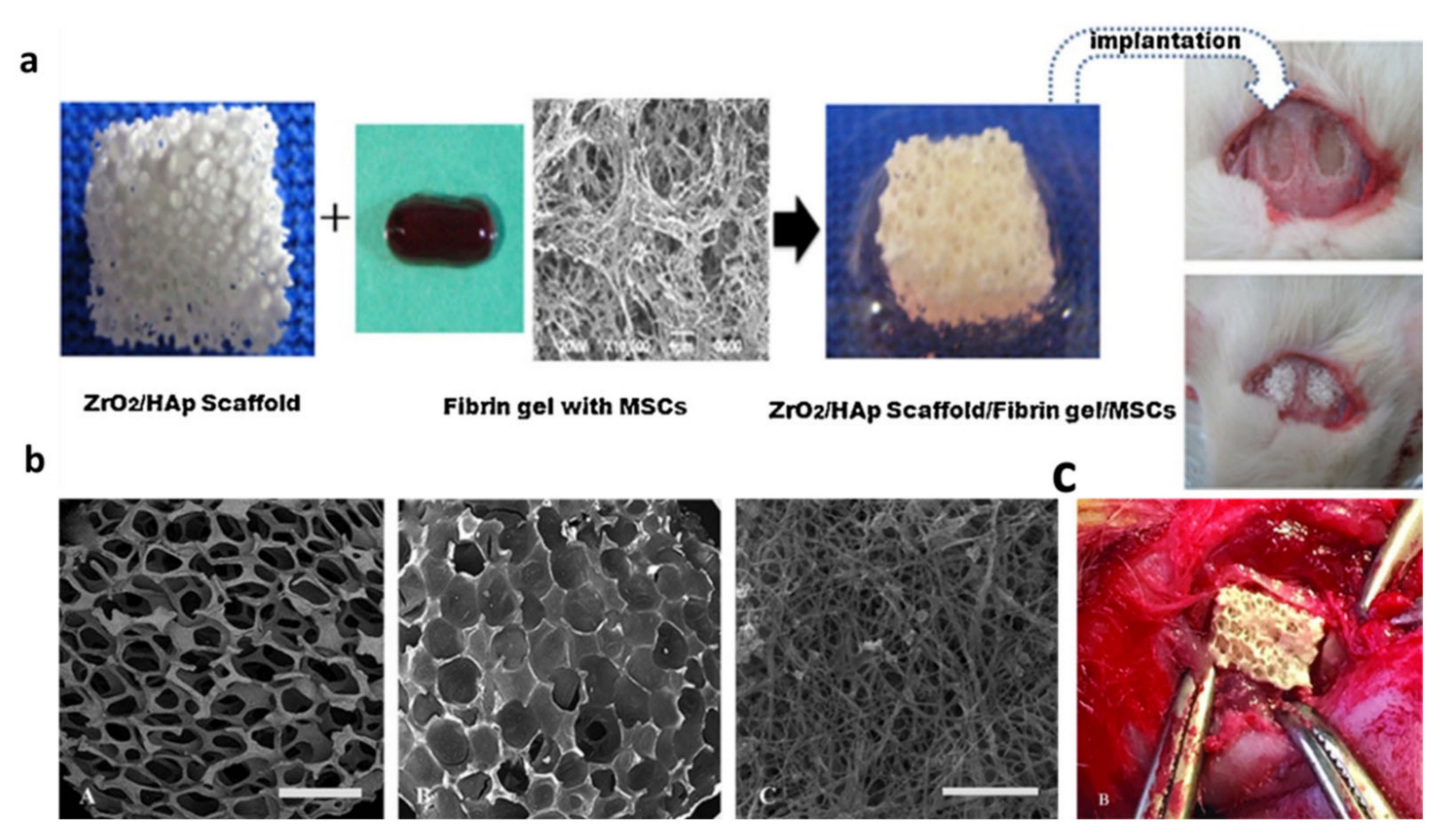
(**a**) Fabrication of the zirconia/HA scaffold via polyurethane foam-scaffold technique and its successful bone-regeneration in eight-week-old male Sprague dawley (SD) rats. Image is used from An et al., reprinted with permission from Elsevier [20], copyright© 2012. (**b**) PRP-impregnated HA/zirconia frameworks: (**A**–**C**) high-resolution SEM outputs of the PRP-impregnated HA/zirconia frameworks. (**c**) In vivo experiment for PRP-impregnated HA/zirconia frameworks; (**B**) in vivo demonstrations via a rectangular bone defect in the rabbit mandible and replacement of the defect using the deliberately designed rectangular scaffolds. Image used from Shahsavari-pour et al. [47], reprinted with permission from Elsevier, copyright© 2018.

Likewise, in 2012, An et al., performed in vivo studies on a fabricated zirconia-HA composite [20]. The authors constructed zirconia/HA scaffolds via the conventional replication (polyurethane foam-scaffold) technique by dipping polyurethane sponge in the zirconia/HA slurries. The authors demonstrated that the compressive strength of the zirconia/HA scaffold increased from 2.5 to 13.8 MPa with an increase in the zirconia content from 50 to 100 wt%. Additionally, the authors found that the biological activity of the zirconia/HA scaffold was superior to that of pristine zirconia alone. More importantly, in vivo examination using fibrin gel comprising bone marrow-derived stromal cells (BMSCs) loaded with a zirconia/HA bioceramic scaffold offered a promising 3D surrounding for BMSC persistence and enriched the bone restoration near the implanted scaffold. The definite fabrication of the zirconia/HA scaffold via the polyurethane foam-scaffold technique and its successful bone regeneration in eight-week-old male SD rats are presented in Figure 1. Thus, An et al., demonstrated that bioinert zirconia can be used as an effective bone-generation material for larger bone defects with the aid of bioactive HA composite formation.

Interestingly, platelet-rich plasma (PRP) was introduced as a bone-growth-supporting agent to HA/zirconia scaffolds by Latifi et al., and Shahsavari-pour et al. [46,47]. In particular, zirconia scaffolds were produced via the polyurethane foam-replication technique and subjected to FA coating and HA coating via the slurry technique, followed by PRP/heparin sulfate (HS) impregnation to obtain HA/zirconia/PRP scaffolds. Scanning electron microscopy (SEM) of the HA/zirconia scaffolds revealed their high porosity, where the pores were impregnated with PRP gel, and the high-resolution SEM outputs of the PRP-impregnated HA/zirconia frameworks revealed nanoscale porosity (Figure 1b). Enriched osteoblastic proliferation and mineralisation of MG-63 cells were observed for the PRP/HS-impregnated scaffold. To examine the bone-restoration capability of the HA/zirconia scaffold with and without PRP, the authors performed an in vivo experiment, creating a rectangular bone defect in a rabbit mandible and replacing the defect using custom designed rectangular scaffolds (Figure 1c). The in vivo studies were conducted for a period of 8 weeks, and the HA/zirconia/PRP scaffolds were found to repair the artificial bone defects. The authors reported that the HA-zirconia scaffolds with and without PRP accurately imitated the bone mandibular properties in the short-term studies. Nonetheless, long-term observations revealed that PRP played no role in enhancing the synergic regenerative properties. It has been proposed that PRP has osteoinduction and antimicrobial activities.

Utilisation of β-TCP as a bioactive composite for tailoring the structural properties of zirconia scaffolds was proposed by Alizadeh et al., in 2014 [48]. In detail, the 3D β-TCP/zirconia (yttrium-stabilised) scaffold composite formation is favoured by external slurry mixing of β-TCP/zirconia at different wt% ratios (zirconia.Y_2_O_3_/β-TCP: A1:50/50, A2:40/60 and A3:30/70) and subsequent sponge formation using a polyurethane sponge (polyurethane foam-replica technique). The authors studied the physical and mechanical outputs with diverse β-TCP/zirconia ratios. They established that the porosity of the β-TCP/zirconia scaffolds can be changed from 65% to 85%. Subsequently, the authors confirmed that the compressive strength varied from 4.95 to 6.25 MPa with an increase in the zirconia content from 30% to 50%. The in vitro biological activity of the β-TCP/zirconia scaffolds was characterised using human endometrial stem cells, and it was found that the cell attachment and proliferation were enriched for the β-TCP/zirconia scaffolds with a ratio of zirconia.Y_2_O_3_/β-TCP 30/70. Conversely, Mohammad et al., reported that reinforcing the HA (75 wt%) matrix with 25 wt% zirconia enhanced the apatite-layer formation on the surface of the porous scaffold and increased the compressive strength to approximately 13.2 MPa [49].

A porous monolithic functionally gradient FG/BCP/zirconia scaffold with a cancellous bone structure was designed via the conventional polyurethane foam-replication technique by Lee et al. [50]. To recover the bioactive properties and reduce the amount of microdefects, the BCP/zirconia and BCP slurries were treated on the monolithic zirconia scaffold as an in-between layer and an external layer, respectively. The dimensions of the knitted pores and supports were approximately 100–250 μm and 110–300 μm after multilayer addition, which were appropriate for bone-renewal. The biological activity of FG/BCP/zirconia was evidenced by the rapid proliferation and cell attachment of the osteoblast-like MG-63 cells.

The use of dual bioceramic β-TCP/HA coatings on a polyurethane foam-replication technique-derived zirconia composite scaffold was described by Song et al. [51], and in vitro cellular-behaviour measurements provided evidence for the MC3T3-E1 pre-osteoblastic cell activity. Compared with titanium, the osteoblast activity on the surface-activated β-TCP/HA/zirconia composite scaffold was higher. Additionally, the osteoblast activity was mainly affected by the microstructure rather than the coating group. The short duration of the cellular-level investigation and the partial distribution of the coating materials in the interconnecting pores were limitations of this study that must be addressed in the future.

**Figure 2:**
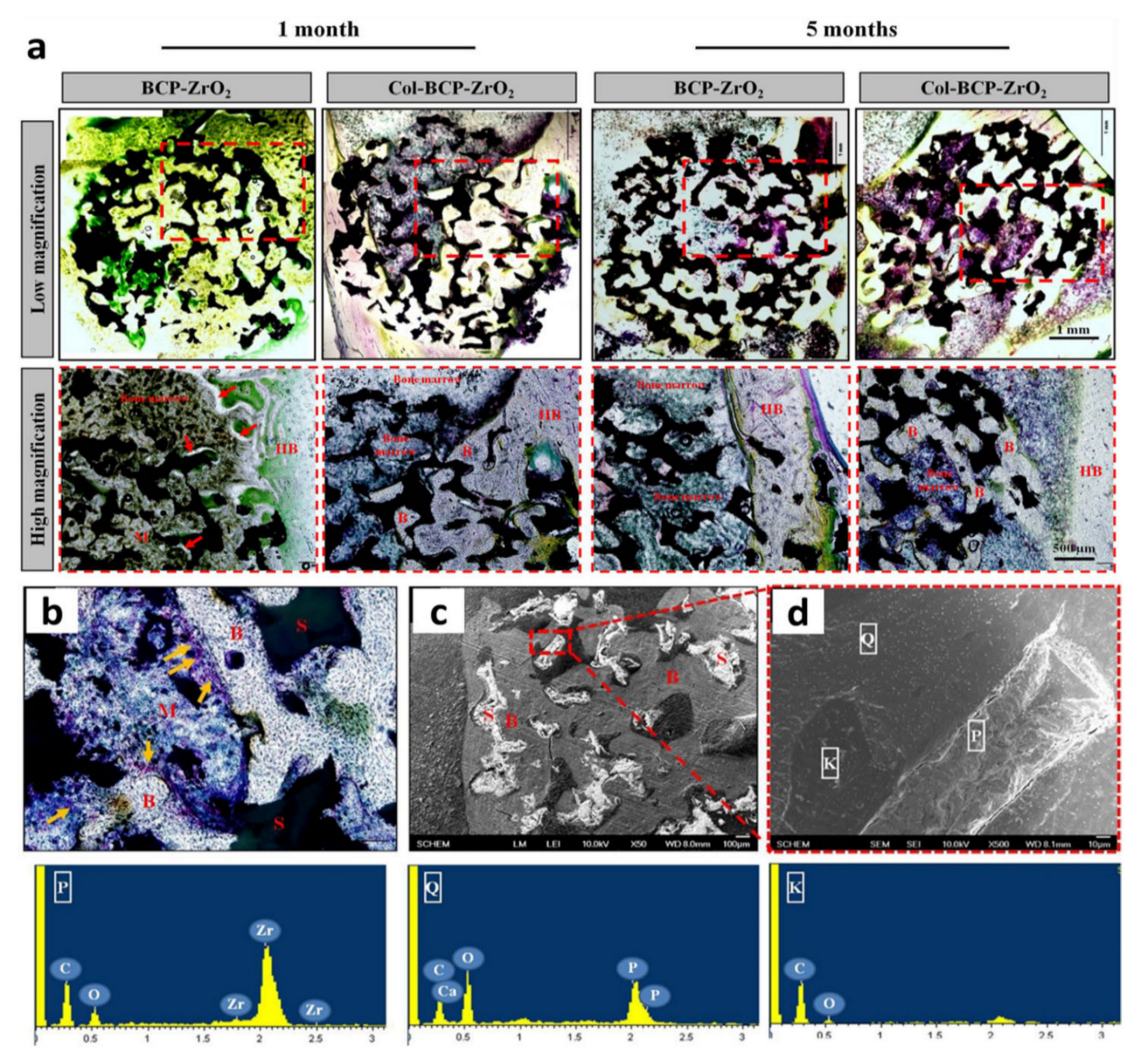
(**a**) Histological analysis of BCP/zirconia and Col-BCP/zirconia scaffolds 1 and 5 months after implantation in the femurs of rabbits, observed via Villanueva bone stains at low and high magnifications. HB: host bone; M: bone marrow; B: new-bone formation. (**b**–**d**) Histological analysis of Col-BCP-zirconia scaffolds after 5 months of implantation in the femurs of rabbits, observed via different methods. (**b**) Villanueva bone stain: HB, host bone; M, bone marrow; B, new-bone formation; S, scaffold. The SEM image in (**c**) is magnified in (**d**). The energy-dispersive X-ray spectroscopy profiles of P, Q and K were taken from (**c**). Images used from Lee et al. [52] reprinted with permission from Elsevier, copyright© 2015.

Furthermore, Lee et al., extended the BCP/zirconia scaffold study to design a unique multilayer BCl/zirconia scaffold with immobilised collagen surface modification (Col-BCP/zirconia) via a polyurethane foam-replication technique [52]. The average pore size of the scaffolds was in the range of 160–500 μm, which was sufficient for inducing new-bone growth [53]. In vitro cell proliferation and differentiation studies (MC3T3-E1 pre-osteoblast cell) revealed that the collagen-modified Col-BCP/zirconia scaffold was superior to the unmodified scaffolds. Collagen inclusion improved the cytocompatibility of the BCP/zirconia scaffold without affecting the bulk properties. More importantly, in vivo examinations of the Col-BCP/zirconia scaffold implanted into rabbit femurs after 1 and 5 months indicated that they have considerable promise for new-bone formation compared with the BCP/zirconia scaffold (Figure 2). Thus, the pioneering research outcomes highlight the importance of the Col-BCP/zirconia scaffold as an artificial bone material.

**Figure 3:**
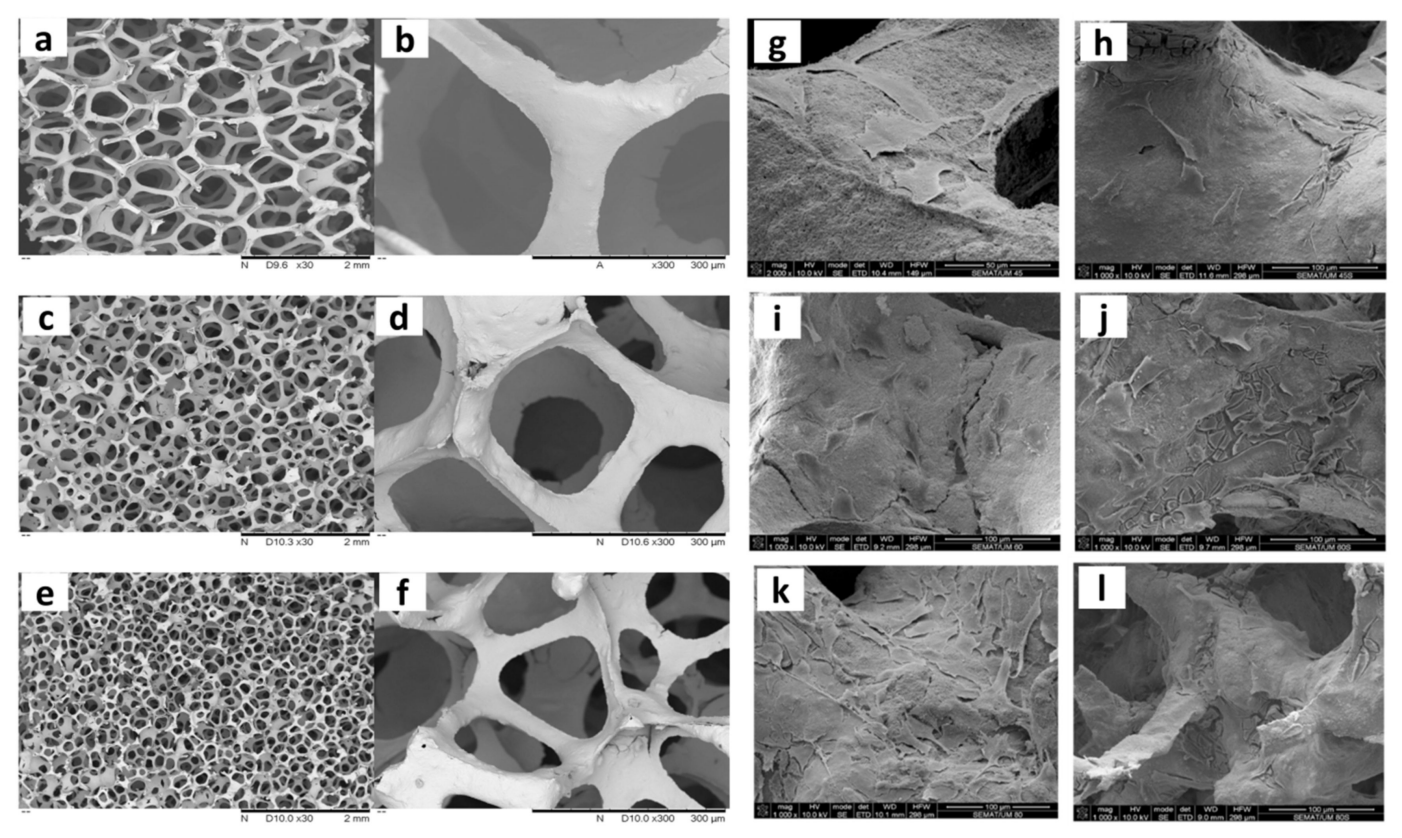
SEM images of 3D zirconia scaffolds (**a**,**b**) 564-Z, (**c**,**d**) 423-Z and (**e**,**f**) 318-Z at low (left) and high (right) magnifications. SEM images of MG-63 osteoblast-like cells on the uncoated 564-Z, 423-Z and 318-Z (**g,i,k**) and coated 564-Z.BG, 423-Z.BG and 318-Z.BG (**h,j,l**) scaffolds after 2 d of cultivation. Images used from Guimaraes et al. [55] reprinted with permission from Elsevier, copyright© 2019.

Additionally, 58S bioactive glass (BG58S)—a combination of calcium/phosphate units and silicate units—was reported to quickly bond with bone and stimulate bone formation, and it has been widely studied for dental implant applications [54]. This highly advantageous feature of BG58S was employed to modify the surface activity of zirconia scaffolds for the first time by Guimaraes et al., in 2019 [55]. Initially, zirconia scaffolds were designed using the conventional polyurethane foam-replication technique, with average pore diameters of 318, 423 and 564 μm, validating the 3D open-cell constructions. The BG58S coating was applied by immersing the zirconia scaffolds in a BG58S sol-gel solution. The coating thickness was controlled by optimising the viscosity of the sol-gel solution and altering the immersion rate, as shown in Figure 3a–f. The authors verified the in vitro biocompatibility of the zirconia scaffolds with and without the BG58S coating by employing MG-63 osteoblast-like cells (Figure 3g–i). The cell feasibility and proliferation were enhanced with a reduction in the pore size. Additionally, the scaffolds with the BG58S coating enhanced the cell viability and promoted cell proliferation, highlighting the importance of the chemical composition on the surface. The results of this study emphasise the significance of the chemical configuration on the surface, aperture diameter and microporosity in the utilisation of zirconia scaffolds as bone grafts.

As mentioned previously, in 2020, Gouveia et al., used the protocol reported by Guimaraes et al., in 2019 for the synthesis of 58S bioactive glass and the fabrication of porous zirconia scaffolds reinforced with 58S bioactive glass [56]. Additionally, polyurethane sponges with 4, 60 and 80 pores per inch were fabricated. Moreover, the authors described an additional processing route that involved coating the pre-sintered zirconia scaffold template (1150 °C) with bioactive glass, followed by sintering at 1500 °C. The infiltration process occurred. Along with this processing step, an additional process was conducted, which involved BG58S coating and heat treatment at 600 °C.

Pristine zirconia grafts were studied for dental implant applications in the early 1980s because of their excellent aesthetic appearance and bioinert properties. However, in the available literature, there are limited research articles on the use of pristine zirconia grafts for bone-repair applications. Zhu et al., (2015) reported the optimisation of porous nanosized zirconia scaffolds via a replica technique with a sufficient porous structure as that of a cancellous bone [57]. The porosity and pore size of the scaffold were controlled by changing the sintering conditions. The tremendous cell adhesion and enhanced proliferation of BMSC cells were observed after 14 d of incubation for a scaffold with 75.2% porosity. Additionally, the scaffold exhibited suitable mechanical properties for load-bearing applications.

Recently, Kim et al., (2018) studied the bone-formation abilities of pristine zirconia grafts engineered using the polyurethane-based sponge replication technique [27]. The designed grafts were subjected to in vivo studies to replace the bone defects in rabbit calvaria and compared with commercial graft materials including Osteon II (Os) and Tigran PTG (Ti). Even though the experimental groups achieved a great extent of new-bone development than compared with the defect group, the differences in the results among the experimental designs were insignificant owing to the similar granule sizes, shapes and porosities of the graft materials. Hence, it is important to design highly porous zirconia scaffolds with a definite bone size and shape using advanced techniques or to alter the surface of zirconia for enhancing the bioactivity.

Askari et al. [58] targeted the advancement of a computational framework (together with experimental confirmation) to regulate the mechanical characteristics of zirconia foams with different pore sizes (manufactured using the foam replica method) for bone-tissue reestablishment applications. Micro-computed tomography (CT) images were filtered to separate noise and smooth margins before fabricating 3D zirconia foams with an adaptive body-centred cubic background framework. The authors verified and scrutinised the stress distributions and magnitudes, scaffold deformation, stresses and plastic strains using the developed micro-CT-based finite-element model. The model was capable of depicting the mechanical stimuli on cells and the confined stress effort in the scaffolds.

### 2.2. Freeze-Drying Technique

Rather than fabricating zirconia in the form of a 3D structure, the 3D bioactive coating layer can be targeted over the zirconia surface. For example, the development of a thick, scaffold-like HA coating on durable zirconia substrates via a freeze-drying-assisted technique for fabricating porous scaffold-like HA/zirconia composites was proposed by Jiang et al. [59]. In vitro tests confirmed that the porous scaffold-like HA/zirconia composites were bioactive. Additionally, the 3D-HA coated onto the core zirconia retained sufficient mechanical properties for load bearing.

The foregoing studies mainly involved the use of calcium phosphate-based bioceramics with bone-like properties to reproduce the bone-graft activities of zirconia scaffolds. However, Teimouri et al., in 2015 established the use of inorganic polyoxometalates (POMs) with a unique biological property to promote the biological activity of zirconia by constructing a POM/zirconia/silk fibroin nanocomposite framework via the freeze-drying method [60]. The in vitro bioactive behaviour of the POM/zirconia/silk fibroin in simulated body fluid (SBF) was examined, and a uniform distribution of fibroblast cells on the POM/zirconia/silk fibroin composite scaffold was observed. The authors introduced a unique method for enhancing the bioactivity of bioinert zirconia; however, in vivo studies must be conducted to observe the effects of inorganic POMs in the human biological environment. The authors performed another study based on silk fibrin (SF) and nano-zirconia; however, the POM polymer was replaced by chitosan (2%) to fabricate a biocomposite scaffold with an interconnected porous structure using a freeze-drying technique [61]. The compression strength and modulus of the composite scaffold were twofold higher than those of the polymer scaffold (SF/CS) owing to the existence of zirconia in the polymer matrix. Moreover, in an evaluation of human gingival fibroblast (HGF) cells, the composite scaffold exhibited higher biocompatibility.

**Figure 4:**
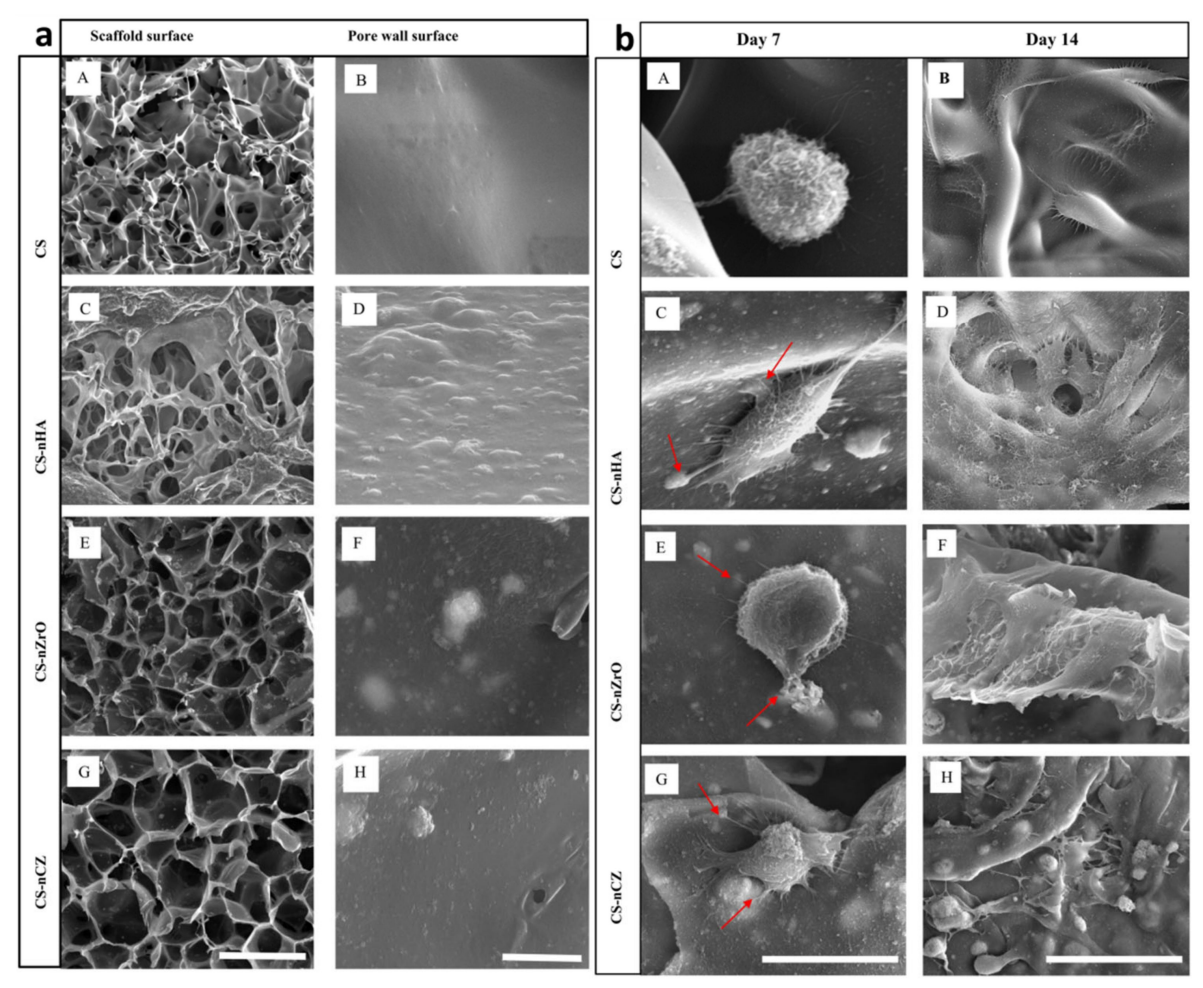
(**a**) SEM images showing the highly porous morphology of the scaffold surface (**A**,**C**,**E**,**G**) and magnified images showing the surface of the scaffold wall (**B**,**D**,**F**,**H**); the scale bars indicate 100 and 2 μm for the scaffold surface and the pore wall surface, respectively. (**b**) SEM images (**A**–**H**) showing the morphology of pre-osteoblast attached and proliferating along the surfaces of different scaffolds; the scale bars indicate 20 and 50 μm in the images taken on days 7 and 14, respectively. Images used from Gaihre et al. [62] reprinted with permission from Elsevier, copyright© 2019.

Recently, a comparative investigation of the physical and biological properties of chitosan-nano-HA (CS-nHA), chitosan-nano-zirconia (CS-nZrO) and chitosan-nano-calcium zirconate (CS-nCZ) porous composite scaffolds (Figure 4a) fabricated via freeze-drying for bone-reestablishment applications was performed by Gaihre et al. [62]. The in vitro activity of the OB-6 pre-osteoblast cells was superior to that of the extended filopodia on CS-nHA and CS-nZrO compared with the CS-nZrO composite scaffolds (Figure 4). The authors expected to investigate further studies on the osteogenic capability of CS-nZrO composite scaffolds.

### 2.3. Pore Former/Space Holder Technique

The vacuum slip casting technique was used to construct an alumina/zirconia composite scaffold with the aid of expanded polystyrene (EPS) beads (acting as pore formers) by Liu et al. [63]. The designed scaffold exhibited homogeneously circulated interconnected pores. The alumina/zirconia composite scaffold was further coated with a thick bioactive glass (58S33C) layer. The bioactive glass-coated alumina/zirconia scaffolds exhibited optimal porosities (60–66%), high strength (5.42–7.52 MPa) and enhanced bioactivity (apatite-layer formation in the SBF solution after 24 h). The authors showcased multiple bioactive glass-coated macropores as permanent scaffolds for bone-tissue restoration.

In one of the reported studies, the bone-growth activities of yttria-stabilised zirconia (YSZ) and magnesium-stabilised zirconia (MgSZ) porous scaffolds were compared (the scaffolds were formed using EPS beads) [64]. The MC3T3-E1 pre-osteoblast cell activity on the YSZ was enriched owing to the surface chemical dissimilarities between the YSZ and the MgSZ. However, the authors claimed that both YSZ and MgSZ porous ceramics can be effective scaffolding materials for cortical bone, because of their comparable mechanical and biological properties.

### 2.4. Solvent Casting and Salt Leaching

Solvent casting and particulate leaching are among the simple and time-effective techniques for producing bioceramic scaffolds with the desired porosity [65]. Unique PLA-HA-YSZ nanocomposite scaffolds with diverse compositions were fabricated via solvent casting and particulate leaching by Ziaee et al. [66]. Among all the ratios tested, a PLA15%—HA-15%YSZ nanocomposite scaffold exhibited the highest compressive strength and SBF activity. The compressive strengths of the scaffolds were reduced after they were soaked in SBF, and the scaffolds containing HA underwent larger strength reductions than those containing YSZ.

In 2018, Mokhtar et al., conducted a case study to assess the efficacy of a custom-made porous zirconia scaffold along with a buccal trapezoidal flap for closure of oroantral fistula [67]. This study was performed on 10 patients suffering from oroantral defects due to extraction of the first and second premolars in a maxillary region with dimensions of approximately 6–9 mm. Initially, a virtual bone model with defects was constructed using the stereolithography photopolymer-based 3D-printing technique from cone-beam computed tomography (CBCT). The zirconia scaffold was prepared via solvent casting and the salt-leaching technique, and the fitness was assessed with the bone model before the sterilisation process. Clinical (extraoral and intraoral examinations) and radiological (CBCT) assessments were performed before and after surgery. Postoperative clinical follow-up evaluations were performed at 2 weeks, 1 month and 3 months. A radiographic examination was conducted after 2 weeks using panoramic radiography. CBCT was performed after 3 months to evaluate the bone formation; the bone density was compared with that during preoperative CBCT. The intensity of pain and frequency of minor complications (postoperative bleeding/edema) were significantly reduced over time, while the bone density increased by approximately 41.2%. The authors found that the zirconia scaffolds with interconnected porous structures enrich the formation of new bone in oroantral defects.

## 3. Fabrication of 3D Zirconia-Based Scaffolds via the Digital Technique

### 3.1. Computer-Aided Design/Computer-Aided Milling (CAD/CAM) Technique

After the acclaimed applications of zirconia ceramics in dental crowns and implants, zirconia ceramics were expected to dominate the biomedical field. However, the direct utilisation of zirconia scaffolds to reconstruct bone is also initiated in the maxillofacial reconstruction area in the dental field. Aftan et al., developed an innovative method for mandibular reconstruction [68]. The authors designed a patient-specific zirconia prosthesis via the CAD/CAM technique using a zirconia block. They employed the zirconia prosthesis technique for the restoration of mandibular flaws in a human trial. This case study was performed on 20 patients for 62 months, with defects caused by mandibular trauma, tumours, and congenital abnormalities, which required surgical resection and reconstruction. A few patients underwent two surgeries; the Boweman Conroyd appliance was placed in the first surgery, and it was replaced by the predesigned zirconia prosthesis in the second surgery. The zirconia prosthesis was designed according to the patient defects and surrounding anatomical structures using a 3D CAD model. Milling was performed on the zirconia block, followed by sterilisation. For the placement of a dental implant, the authors voluntarily created a hole in the prosthesis. The results were encouraging, with a 95% success rate, and the reconstruction did not affect the mandibular function or aesthetics. Minor complications (pain and edema) were reported.

**Figure 5:**
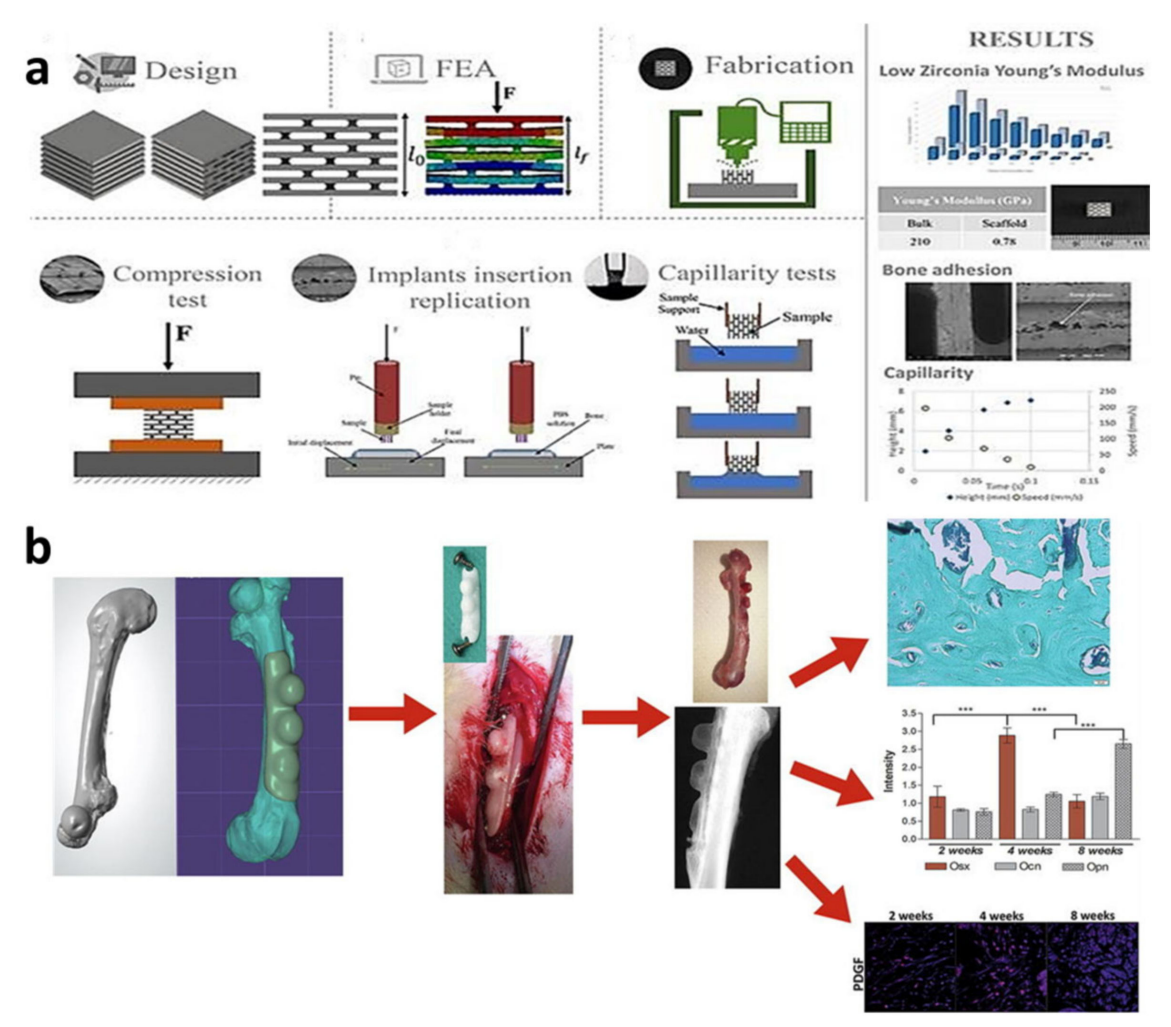
(**a**) Schematic of zirconia scaffolds designed via a CAD/CAM model. Image used from Marques et al. [69] reprinted with permission from Elsevier, copyright© 2019. (**b**) Detailed illustration of the design and milling of 3D-tailored CAD/CAM zirconia space-maintaining strategies for a rat femur. Image used from Tetre et al. [70] reprinted with permission from Elsevier, copyright© 2021.

It is well known that CAD/CAM is a destructive technique that cannot produce zirconia scaffolds with high porosity. Recently, an interesting approach for designing zirconia scaffolds with ~40% porosity and a multilayer assembly via CAD/CAM was proposed by Marques et al. [69]. The distinctive design strategy involves 5-axis milling (XYZ axes and rotating axes (A and B axes)) that allows the part to move 360° in both directions and 20° towards the front and back [69]. A schematic of the zirconia scaffolds designed using the CAD/CAM model is presented in Figure 5a. The scheme comprehensively illustrates the process of designing complex zirconia scaffolds using a modified CAD/CAM model, followed by mechanical testing (compression testing), whereby the Young’s modulus values were found to be associated with the host bone. The rapid diffusion behaviour of water inside the channels of the zirconia scaffolds was established using capillary testing. Likewise, to replicate the endosseous implant, the authors demonstrated implant insertion imitation practice, wherein the implant exhibited superior fixation at the early stage of implantation. Tribological tests revealed that the cavities/valleys inside the scaffolds were loaded by bone. Even though the authors mimicked the bone implantation of the zirconia scaffolds via tribological tests, it is essential to verify the cell activity of the scaffolds in the biological environment.

To all the above, most recently, Tetre et al., revealed a unique method for evaluating bone reconstruction using zirconia scaffolds in the rat femur via a 3D-CAD/CAM approach [70]. A thorough illustration of the design and milling of 3D-tailored CAD/CAM zirconia space-maintaining strategies for a rat femur is presented in Figure 5b. As shown, a 3D-customised assembly was produced with different heights. The authors verified the guided bone regeneration (GBR) abilities of the rat femur via Gomori’s trichrome histomorphometrical examination. The authors monitored GBR activation at different time intervals (2, 4 and 8 weeks) and found that the Haversian system was present in freshly grown bone. Thus, the tailored milled zirconia scaffold in this study provided a detailed understanding of progressive bone-tissue construction. The successful bone-reconstruction ability of the zirconia scaffold highlights the effectiveness of the 3D-tailored CAD/CAM technique for extending its application to various orthopaedic applications.

### 3.2. Extrusion-Based Techniques

#### 3.2.1. Multi-Pass Extrusion Technique

**Figure 6:**
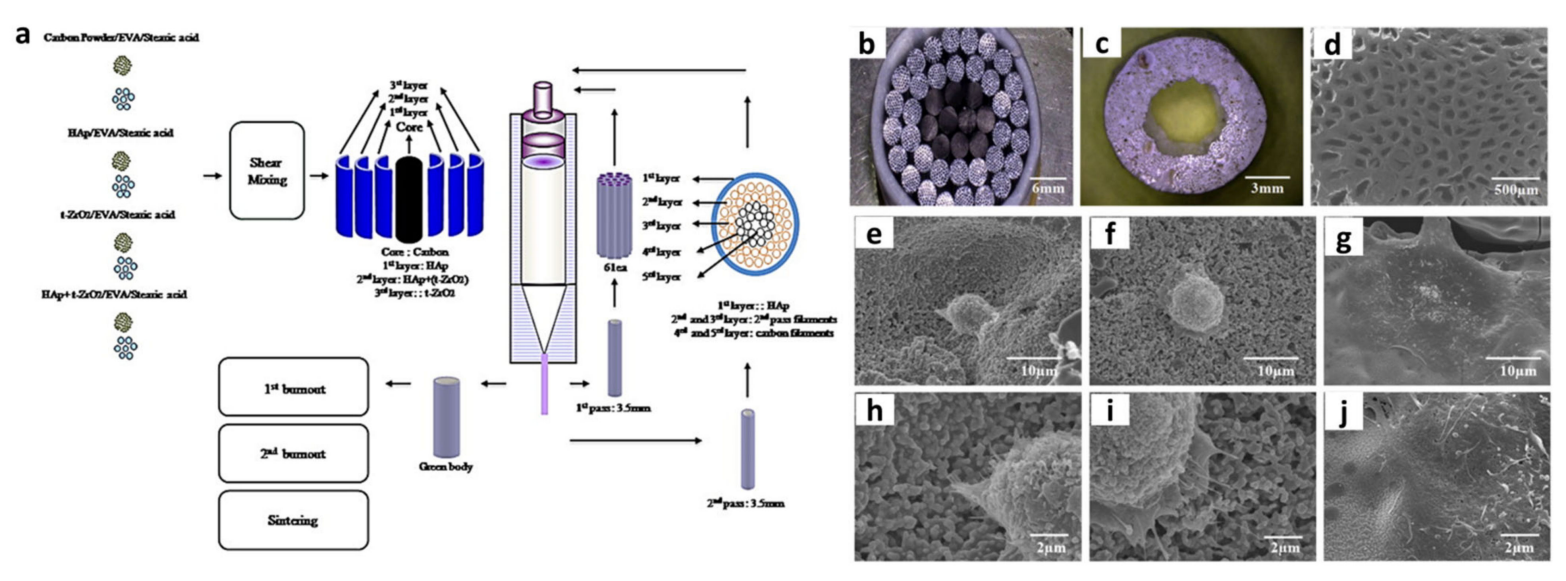
(**a**) Schematic of the multi-pass extrusion process. Photographs taken before extrusion and after sintering (**b**) arranged filaments and shell before final (3rd) extrusion, (**c**) sintered bodies at 1500 °C, (**d**) enlarged SEM image of the pores. SEM images of MG-63 cell morphologies on TCP/TCP-(t-zirconia)/t-zirconia composites at incubation times of 15 min (**e**,**h**), 30 min (**f**,**i**) and 60 min (**g**,**j**). Images used from Lee et al. [76] reprinted with permission from Elsevier, copyright© 2011.

Following the clinical success of an extrusion-based technique to fabricate zirconia scaffolds for dental applications, the design of 3D zirconia scaffolds for bone-regeneration applications has been widely studied [23]. Lee et al., analysed this technology even before the worldwide upsurge in zirconia scaffolds for dental applications [71]. They fabricated HA-coated micro-channelled fibrous Al_2_O_3_-(monoclinic, M-zirconia)/(tetragonal, t-zirconia) composite scaffolds via the multi-extrusion process in 2004 [72]. In a preliminary study, they developed a multi-extrusion printing technique for designing zirconia composite scaffolds with good mechanical properties. In another study, the research group varied several printing parameters, including the pore-gradient rate, extrusion ratio and microstructure, to optimise the experimental conditions for the fabrication of Al_2_O_3_-zirconia composite scaffolds [73]. Their fabrication technique involved the mixing of bioceramics using the polymer ethylene vinyl acetate, followed by extrusion. The extrusion process is repeated to build a constantly porous core assembly with alternating Al_2_O_3_ and zirconia layers. Using this custom design, the authors constructed various scaffolds (both individual scaffolds and binary mixtures) over the subsequent years to achieve unique interconnected architectures [71,74,75]. Later, in 2006, they demonstrated the in vitro understanding of Al_2_O_3_-(M-zirconia)/t-zirconia composite scaffolds with well-established 3D-interconnected micropores using human osteoblast-like MG-63 cells. The MG-63 cells were attached on the rough outer layer as well on the micropores inside the scaffold body [75]. In the following years, owing to the repeated efforts of Lee et al., various structural advantages have been implemented, yielding diverse zirconia composite scaffolds that are useful for bone-regeneration applications. For example, using the multi-extrusion process, Lee et al., constructed bone-like continuously porous TCP/TCP-(t-zirconia)/t-zirconia composites (originally, they used HA that transformed into TCP during heat treatment) (Figure 6a) [76]. To rectify the microcracks upon sintering due to thermal expansion mismatch issues, the authors made the boundary amongst the pores with three types of laminates (Figure 6b–d). Cross-sectional SEM study confirmed the uniform pore channels and the absence of delamination and cracks after the sintering process. The Haversian channel dimension and outer cortical sample size were linked at diverse sintering temperatures, and their values were recognised to be 86 μm, 10.3 mm and 53 MPa, respectively. Furthermore, complete adhesion and distribution of osteoblast-like MG-63 cells were observed in in vitro survival studies of the artificial cortical bone developed by the authors (Figure 6e–j).

**Figure 7:**
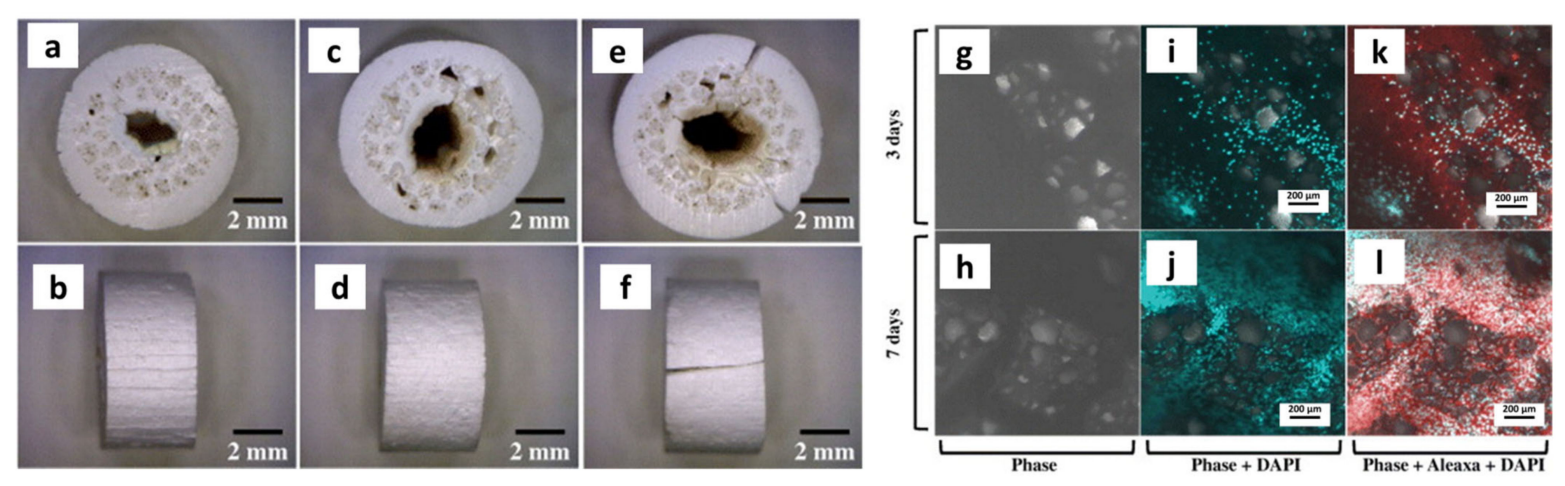
Photographs of HAp/t-zirconia composites sintered at 1300 °C (**a**,**b**), 1400 °C (**c**,**d**) and 1500 °C (**e**,**f**). Confocal microscopy images of osteoblast cell proliferation on an HAp/t-zirconia composite scaffold sintered at 1400 °C for 3 days (**g,i,k**) and 7 days (**h,j,l**). Reprinted with permissionfrom Lee et al. [77], copyright© 2012.

The combined mechanical and biological outputs of the TCP/TCP-(t-zirconia)/t-zirconia scaffolds in this study were proven to be an ideal composite favourable for synthetic cortical bones such as toe joints and finger replacements. This was followed by the authentic demonstration of TCP/TCP-(t-zirconia)/t-zirconia scaffolds as an artificial cortical bone. Lee et al., constructed porous multilayer HA/t-zirconia scaffolds via a multi-extrusion process comprising concentric laminated structures and micro-channelled groups [77]. Microstructural studies revealed that the HA/t-zirconia scaffold consisted of alternating units of HA, HA/t-zirconia and t-zirconia. The mechanical properties of the scaffolds varied significantly with the increasing temperature. The scaffold sintered at 1400 °C exhibited a high compressive output (20 MPa), and no cracks were observed, whereas the sample sintered at 1500 °C exhibited a noticeable crack (Figure 7a–f).

The Haversian passage and interior dimensions were calculated to be 80 μm and 1.9 mm, which were favourable for cell development. Furthermore, in vitro osteogenesis was confirmed by the uniform proliferation of osteoblast-like MG-63 cells (Figure 7g–l). Additionally, the multi-extrusion methodology was employed by Lee et al., to construct a macroporous channelled scaffold comprising a thin zirconia covered with a sacrificial background layer made of PCL/BCP, which undergoes degradation to promote new-bone formation [78]. The zirconia-PCL/BCP multilayer scaffold with the unidirectional cylindrical channels exhibited 78% porosity, along with an acceptable compressive strength (12.7 MPa). The in vitro proliferation of osteoblast-like MG-63 cells inside the pore wall of the zirconia-PCL/BCP multilayer scaffold was confirmed by SEM. Unfortunately, there is not much acknowledgement of Lee et al.’s advancements. We take this opportunity to provide a comprehensive summary of their research results. This will provide readers with a better understanding of the evolution and progress of 3D zirconia scaffolds, promoting their use as standard materials for bone-tissue engineering.

#### 3.2.2. 3D-Bioplotter Technique

**Figure 8:**
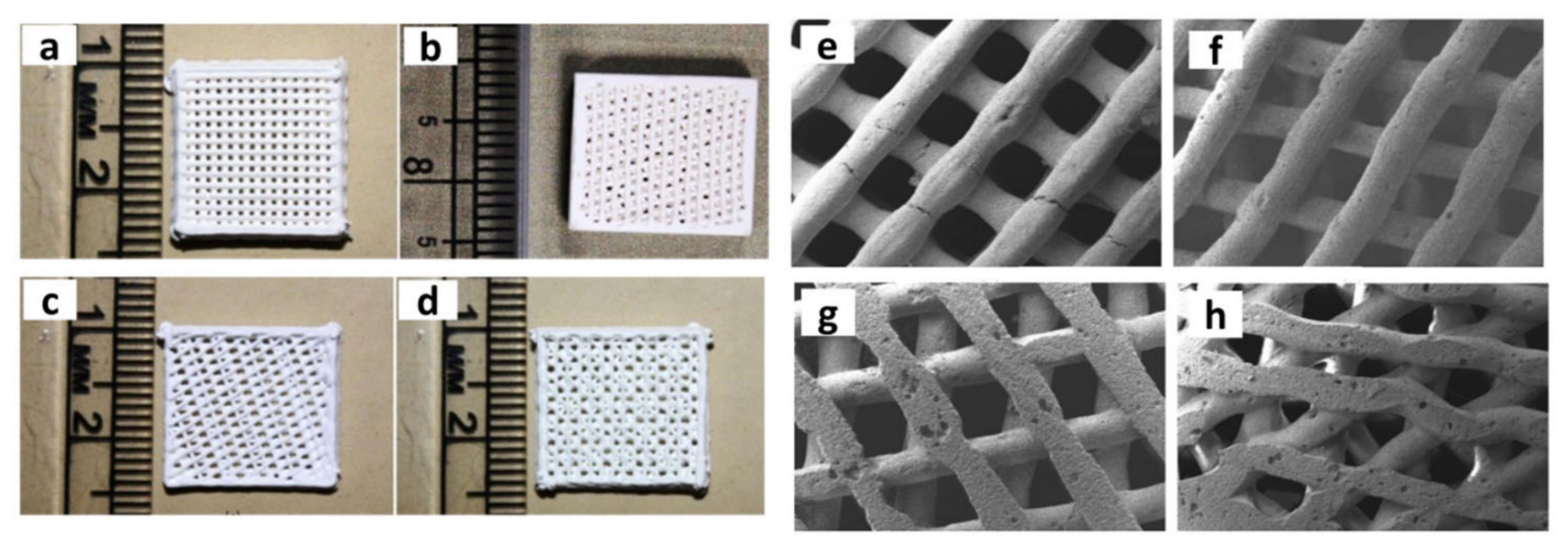
Photographs (**a**–**d**) and SEM images (**e**–**h**) of Sintered β-TCP/zirconia scaffolds with different laydown patterns and (**a**,**e**) 0°–90°, (**b**,**f**) 0°–72°–144°–36°–108°, (**c**,**g**) 0°–108°–216° and (**d**,**h**) 0°–45°–90°–135°, respectively. Reprinted with permission Images used from Sapkal et al. [79], copyright© 2017.

Sapkal et al., used the extrusion-based technique and 3D-Bioplotting to construct 3D β-TCP/zirconia composite scaffolds with diverse architectures for healing large bone deficiencies [79]. Scaffolds (0°–90°, 0°–45°–135°–180°, 0°–108°–216° and 0°–72°–144–36°–108°) tactically designed by the authors with diverse filament alignments (Figure 8a–h) were systematically subjected to mechanical and biological testing evolutions. The in vitro bioactivity capability of the β-TCP/zirconia composite scaffold to replicate the human-bone environment was assessed using MG-63 human osteosarcoma cells. Among the samples tested, the β-TCP/zirconia composite scaffold with the 0°–72°–144°–36°–108° filament orientation exhibited the best mechanical strength and cell-breeding capability.

Moreover, they concluded that the β-TCP/zirconia composite with a 70/30 ratio is favourable for large-size bone-repair applications. The authors proposed future research directions to extend their research to the next level, i.e., the insertion of Haversian canals inside the β-TCP/zirconia composite scaffolds, for imitating blood vessels and stimulating vascularisation.

**Figure 9:**
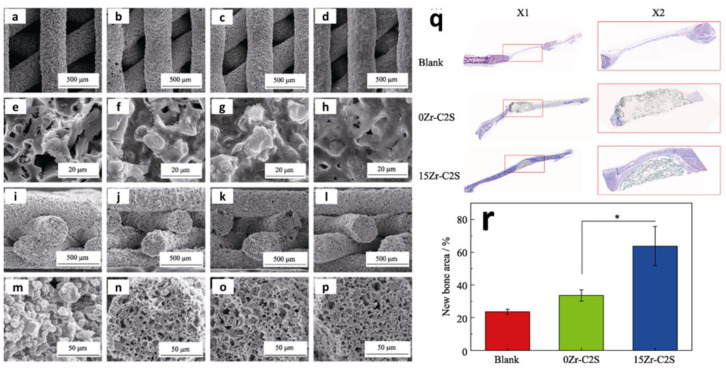
(**a**–**p**) SEM images of (**a**) 0Zr-C2S, (**b**) 5Zr-C2S, (**c**) 10Zr-C2S and (**d**) 15Zr-C2S scaffolds, with SEM fracture surface images from (**i,m**) to (**i**–**p**). In vivo results for 0Zr-C2S and 15Zr-C2S scaffolds (**q**) histological and (**r**) quantitative analysis results for newly formed bone. Reprinted with permission from Fu et al. [80], copyright© 2019.

Fu et al., highlighted the importance of zirconia reinforcement in β-Ca_2_SiO_4_ scaffolds for bone-tissue engineering constructed via the 3D printing of silicone resin-loaded CaSiO_3_/zirconia fillers using the extrusion-based 4th Bioplotter [80]. The amount of zirconia incorporated into CaSiO_3_ was varied from 0 to 15 mol%, and the scaffolds were denoted as (0Zr-C2S, 5Zr-C2S, 10Zr-C2S and 15Zr-C2S). The β-Ca_2_SiO_4_/zirconia scaffolds designed in this study exhibited a rough surface and micropores, as indicated by the SEM results in Figure 9a-p, which are important features for mimicking bone behaviour. The engineered β-Ca_2_SiO_4_/zirconia scaffolds with a woven macropore network exhibited a high porosity (~67%) and compressive strength (~6.1 MPa) favourable for load-bearing bone reconstruction. The enhanced biological activity of the 15Zr-C2S scaffolds in the in vitro rat bone marrow mesenchymal cells encouraged the authors to examine the new-bone formation capability in a real biological environment (in vivo studies). A histological examination revealed that the bone restoration was more pronounced in 15Zr-C2S scaffolds than in a pure β-Ca_2_SiO_4_ scaffold implanted in rat deficiency zones (Figure 9q,r). In summary, zirconia incorporation induces dual properties to β-Ca_2_SiO_4_ scaffolds (i.e., improved mechanical strength and controlled degradation of Ca and Si), which is essential for renewed bone growth.

#### 3.2.3. Fused Deposition Modelling (FDM) Technique

**Figure 10:**
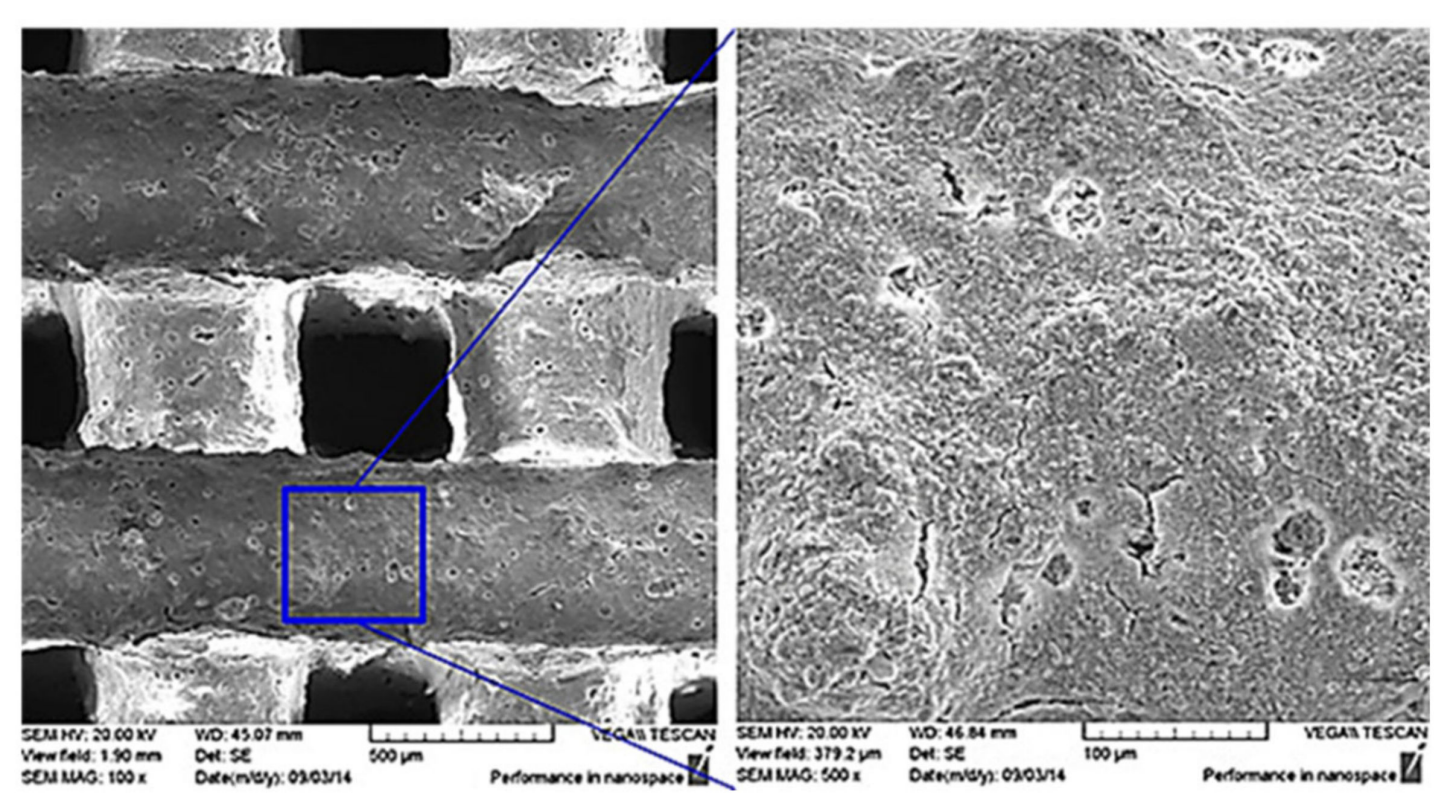
SEM images of the BCP/zirconia scaffold. The left image shows a pore size of approximately 350 µm and printed lattice width of 500 µm. The right side depicts a granular surface of the 3D-printed scaffold after printing and sintering. Images used from Sa et al. [81] reprinted with permission from Willey and sons, copyright© 2018.

Sa et al., fabricated reinforced BCP scaffolds with different amounts of zirconia (BCP/zirconia) in 2018 via the FDM 3D-printing technique [81]. A photograph of the FDM process employed by Sa et al., to design zirconia-reinforced BCP scaffolds. SEM of the BCP/zirconia scaffold with overall dimensions of 6.0 × 6.0 × 3.0 mm^3^ revealed that the pore size was 350 μm (Figure 10). Incorporation of 10% wt% zirconia powder into the BCP scaffolds enhanced the compressive strength without degrading the bioactivity. The authors verified the in vitro bioactivity of the BCP and BCP/zirconia scaffolds in the presence of human mesenchymal stem cells (hMSCs) by comparing the fluorescence staining of the hMSCs with BCP and BCP/zirconia scaffolds. The BCP/zirconia scaffolds exhibited improved expression of bone morphogenic protein-2 compared with the pristine BCP scaffolds. The synergistic effects of zirconia-stimulated scaffolds in achieving an active culture upon osteogenic differentiation of bone tissues were proposed by Sa et al.

In addition to the traditional bioceramics, biopolymers have been investigated for bone-regeneration applications because of their easy processability and controlled degradation amount.

Polycaprolactone (PCL) is the most widely studied biopolymer because of its poor mechanical properties and poor hydrophilicity. PCL alone cannot be used directly as a bone-tissue framework. Hence, similar to most of the bioceramic designs, researchers have attempted to include zirconia to enhance the mechanical properties of PCL [82]. For instance, Wang et al. [83] embedded nano-zirconia with different weight ratios (5, 10 and 20 wt%, i.e., PZ5, PZ10 and PZ20, respectively) into PCL (PCL/zirconia) through the melt mixing method and fabricated ordered scaffolds using the extrusion-based 3D-printing technique. They have found that the pristine PCL, the PCL/zirconia scaffolds exhibited improvements of 50% and 40% in the compressive strength and Young’s modulus, respectively. Furthermore, the improved hydrophilicity and better water-adsorption ability of the PCL/zirconia scaffold are conducive to cell bonding and nutrient passage. For instance, an in vitro bioactivity survival test of PCL/zirconia scaffolds using the MC3T3 cells via laser confocal microscopy revealed that the cell growth rates of the PCL and composite scaffolds (PZ5, PZ10 and PZ20) at 1, 3 and 7 d were >90%. The PZ20 scaffold exhibited superior MC3T3 cell distribution, and the degree of cell proliferation varied with respect to the zirconia content. Thus, the strategic design of PCL/zirconia scaffolds proposed by Wang et al., confirmed the effectiveness of zirconia incorporation in transforming the PCL into a potentially feasible scaffold for bone-tissue restoration.

#### 3.2.4. Robocasting/Direct Ink Writing (DIW) Technique

Robocasting, i.e., DIW, techniques allow the design of high-quality complex ceramic structures via optimal formulation and deposition of colloidal ceramic ink/paste that flows through the printing nozzle with a high solid loading content to form a 3D construct with open porosity and are widely used owing to their cost-effectiveness and easy processing [84].

Few attempts to fabricate zirconia scaffolds using robocasting for replacing bone defects have been reported. The formulation of a zirconia suspension with high solid loading and the optimal rheology plays a crucial role in printing; thus, Brazete et al., investigated the dispersibility of zirconia powder (45 vol%) in a homogeneous aqueous suspension with different ratios (0.3%, 0.5%, 0.8%) of anionic dispersant (Dolapix CE 64) [85]. With 0.3 wt% dispersant, the suspension exhibited shear-thinning rheological behaviour. Furthermore, the authors deliberated the optimised 0.3 wt% dispersed ink with higher zirconia loading of 48 vol% for robocasting.

In 2015, Li et al., reported the fabrication of 3D cylindrical and woodpile-like 3D zirconia scaffolds with interconnected scaffolds via the DIW technique [23]. The DIW design proposed by the authors comprises three major parts: the computer-aided system (CAD), extrusion needle and X-Y-Z platform. They unlocked the access to design 3D zirconia tissue engineering scaffolds with water-based zirconia ink having a zirconia content of 70 wt%. The zirconia ink was printed into layer-by-layer ordered 3D zirconia scaffolds. The compressive strength of the 3D zirconia scaffolds (10 MPa) was higher than that of the HA scaffolds. The in vitro proliferation of HCT116 cells on the 3D zirconia scaffolds was verified using a microscope. Thus, the authors successfully proposed the possibility of the DIW method to design scaffolds with precise control of the porosity for advanced bone-tissue engineering applications.

**Figure 11:**
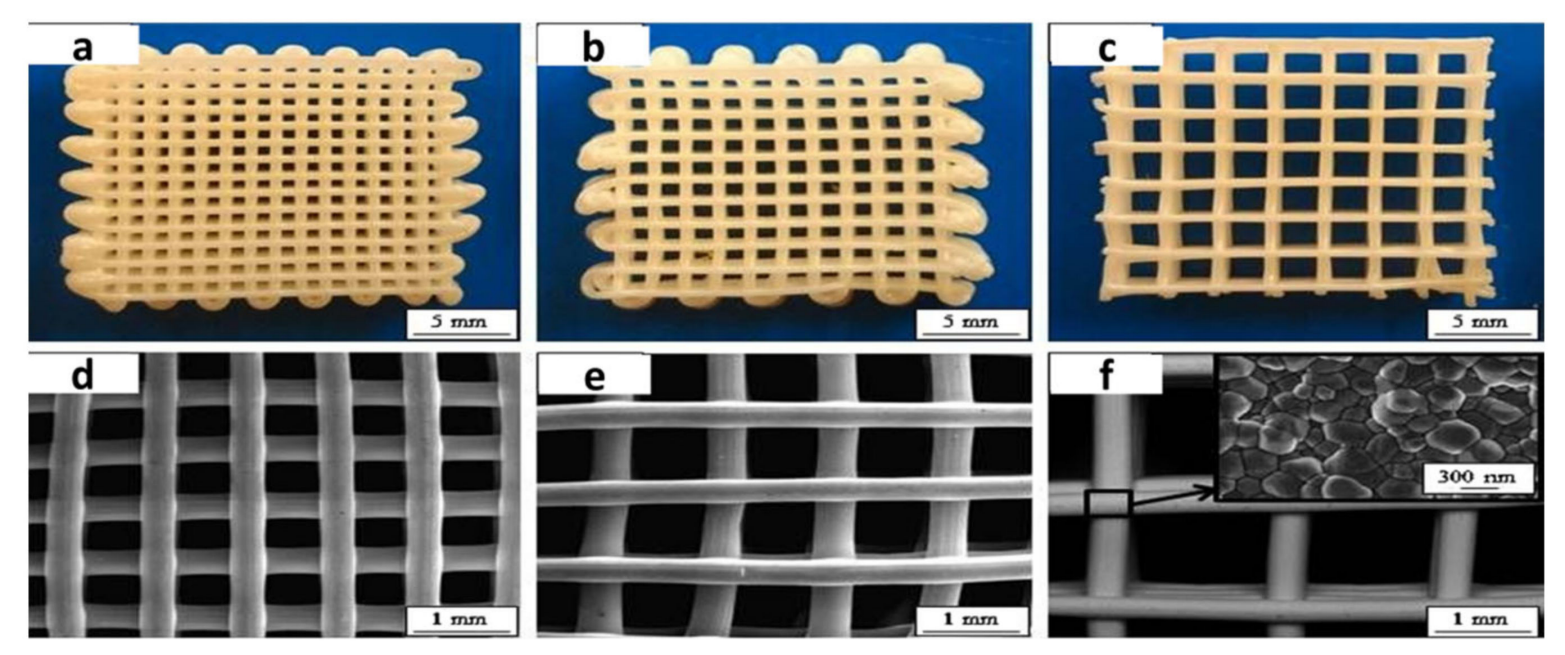
Optical images of 3D periodic zirconia scaffolds with different minimum separation distances between rods (**a**) 0.7 mm, (**b**) 0.9 mm and (**c**) 1.8 mm and corresponding top-surface images (**d**–**f**), respectively. The inset SEM image shows the high degree of sintering achieved within the filaments. Image used from Kocylo et al. [86] reprinted with permission from Elsevier, copyright© 2021.

Recently, Kocylo et al. [86] engineered 3D zirconia scaffolds with 61 and 75.3 vol% porosity via the direct ink-writing method. The SEM results in Figure 11 confirm the uniformity of the zirconia scaffolds with good control in thread and pore openings. With changes in the porosity level, the compressive strength of the printed zirconia scaffolds varied between 20.8 and 62.9 MPa. The authors applied multi-surface alteration tactics involving a dual apatite FA/HA coating to the 3D zirconia scaffolds for obtaining zirconia/FA/HA composite scaffolds (originally proposed by Kim et al. long ago for 3D zirconia scaffolds). For comparison, they applied the HA/HA coating to the 3D zirconia (zirconia/FA/HA scaffolds). The authors performed an in vitro investigation to understand the bioactivity and dissolution properties of the zirconia/FA/HA and zirconia/FA/HA composite scaffolds, which were immersed in SBF and a physiological saline solution (0.9%, NaCl) for 28 d. The zirconia scaffolds with the FA/HA coating exhibited enriched bioactivity and slower dissolution than the zirconia scaffolds with the HA/HA coating. In contrast to other reported studies, Stanciuc et al., developed a new platform with the processing, structural characterisation and interaction of human primary osteoblast cells of a robocast ZTA scaffold [87].

### 3.3. Photopolymerisation-Based Techniques

Since the introduction of photopolymerisation (PP) based 3D printing by Hull in 1986 [88], it has been claimed that PP-based practices have considerable potential for producing 3D bioceramic scaffolds with high accuracy [89].

#### 3.3.1. Digital Light Processing (DLP)

DLP technology has been widely studied for the fabrication of ceramic parts, as it can produce complex ceramic structures with a high precision, fast processing and a high accuracy compared with other 3D-printing technologies. The slurry suspension was prepared by optimising the solid loading of the ceramic, organic monomer, dispersant and photoinitiator, and the slurry was irradiated with ultraviolet light. The rheological property of the ceramic suspension is a key factor in the 3D-printing process and affects the quality of the final product. However, the use of DLP technology in the biomedical field has been restricted owing to the incorporation of the photosensitive resin and photoinitiator which is known to be cytotoxic, into the slurry. Therefore, successive de-binding and sintering processes must be developed before this scaffold can be employed for bone-tissue regeneration [90].

**Figure 12:**
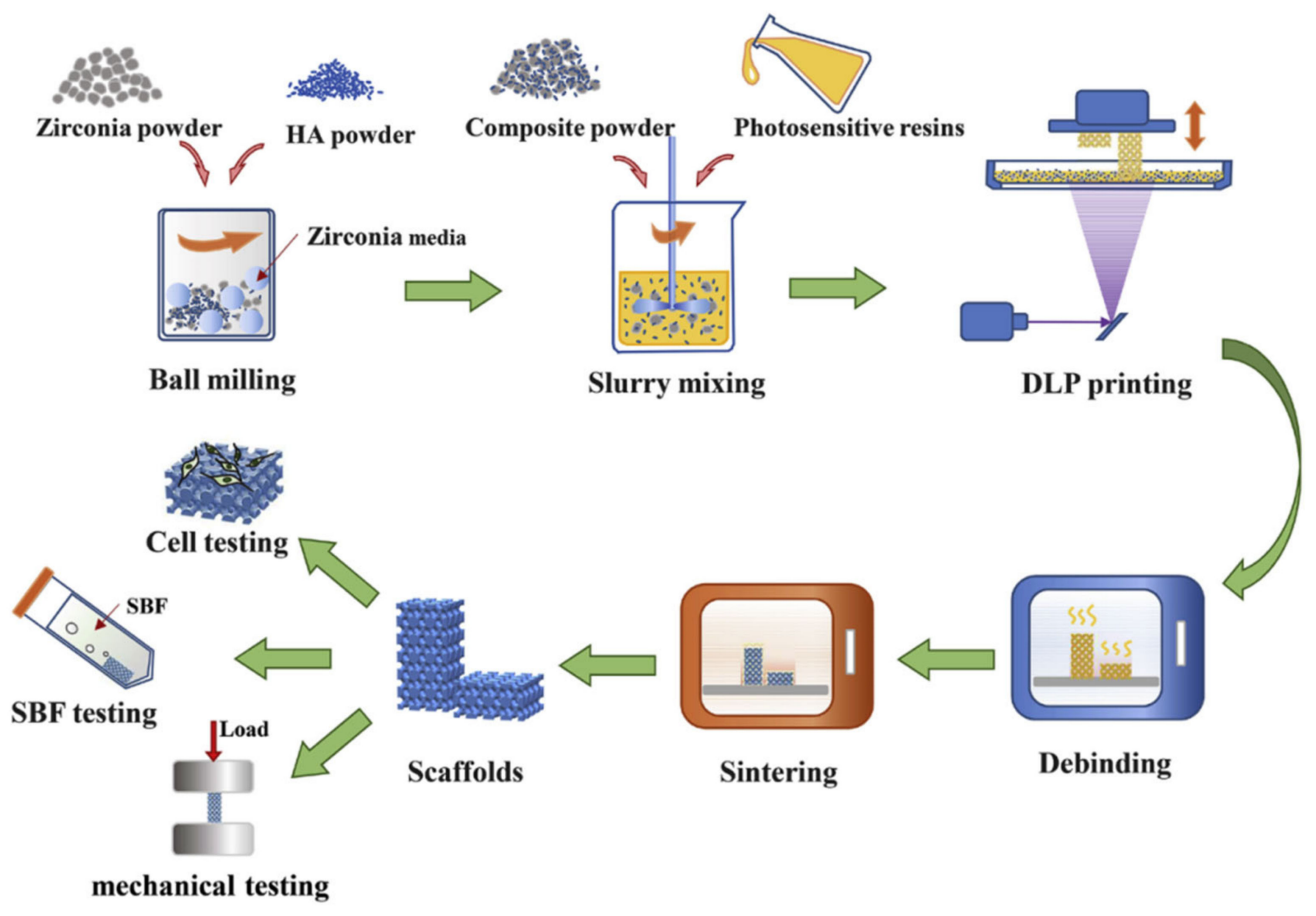
Illustration of the fabrication and testing process. Image used from Cao et al. [91], reprintedwith permission from Elsevier, copyright© 2020.

Few in vitro investigations of zirconia-based scaffolds fabricated using the DLP technique have been reported. In 2020, Cao et al., evaluated the biological activity and mechanical strength of a zirconia/HA porous scaffold fabricated via DLP (Figure 12) [91]. They added HA at various concentrations (0%, 10%, 20% and 30%) to zirconia to enhance the bioactivity of the porous scaffold with partial degradation. With the increasing HA content, the degrees of cell proliferation and differentiation of MC3T3-E1 cells increased, while the compressive strength decreased. However, HA10% exhibited improved strength compared with the control (zirconia) scaffold.

In contrast, Zhang et al., fabricated a porous zirconia toughened HA composite scaffold for bone-tissue engineering using the DLP 3D-printing process with HA as the primary ratio of the scaffold; additionally, a small amount of zirconia was used as a structural stabiliser [26]. The ceramic powder (60 wt%) with a high solid loading into the suspension by modifying the surface of the HA nanoparticles with two organic modifiers (KH570 and oleic acid) before adding to the acrylic resin and for improved dispersion was proposed. Castor oil phosphate was added to reduce the viscosity of the ceramic suspension. The authors reported that the incorporation of zirconia into HA reduced the decomposition of the HA phase and promoted densification during the firing process. The de-binding and sintering of the printed scaffold were performed in a vacuum high-temperature furnace to avoid the generation of internal cracks and defects in the sintered ceramic parts, which are caused by the internal pressure created inside the samples during sintering. The mBMSC exhibited no cytotoxicity in the 3 wt% zirconia group and exhibited increased cell proliferation over a period of time.

#### 3.3.2. Selective Laser Sintering (SLS) Technique

SLS is a well-established additive-manufacturing technique that employs laser energy as a primary source to fuse powder particles into the desired 3D architecture [92]. It has been widely studied in the field of biomedical applications for the fabrication of 3D structures. Shuai et al., for the first time attempted to fabricate a nano-zirconia-reinforced calcium silicate (CaSiO_3_) porous scaffold (CaSiO_3_/nano-zirconia scaffold) via the SLS technique for bone-recreation applications with improved mechanical properties [93]. The authors found that increasing the amount of nano-zirconia resulted in superior mechanical properties. However, when the zirconia concentration exceeded 30%, the zirconia affected the sintering, caused unsolicited agglomeration and degraded the materialising ability. An in vitro analysis revealed that the CaSiO_3_/nano-zirconia (20%) scaffolds had the ability to mock bone features by forming a bone-like apatite layer and promoting superior assembly of MG-63 cells. The authors concluded that the porous scaffolds with the addition of 20% of nano-zirconia exhibited good biocompatibility and mechanical properties.

### 3.4. Electrospinning

**Figure 13:**
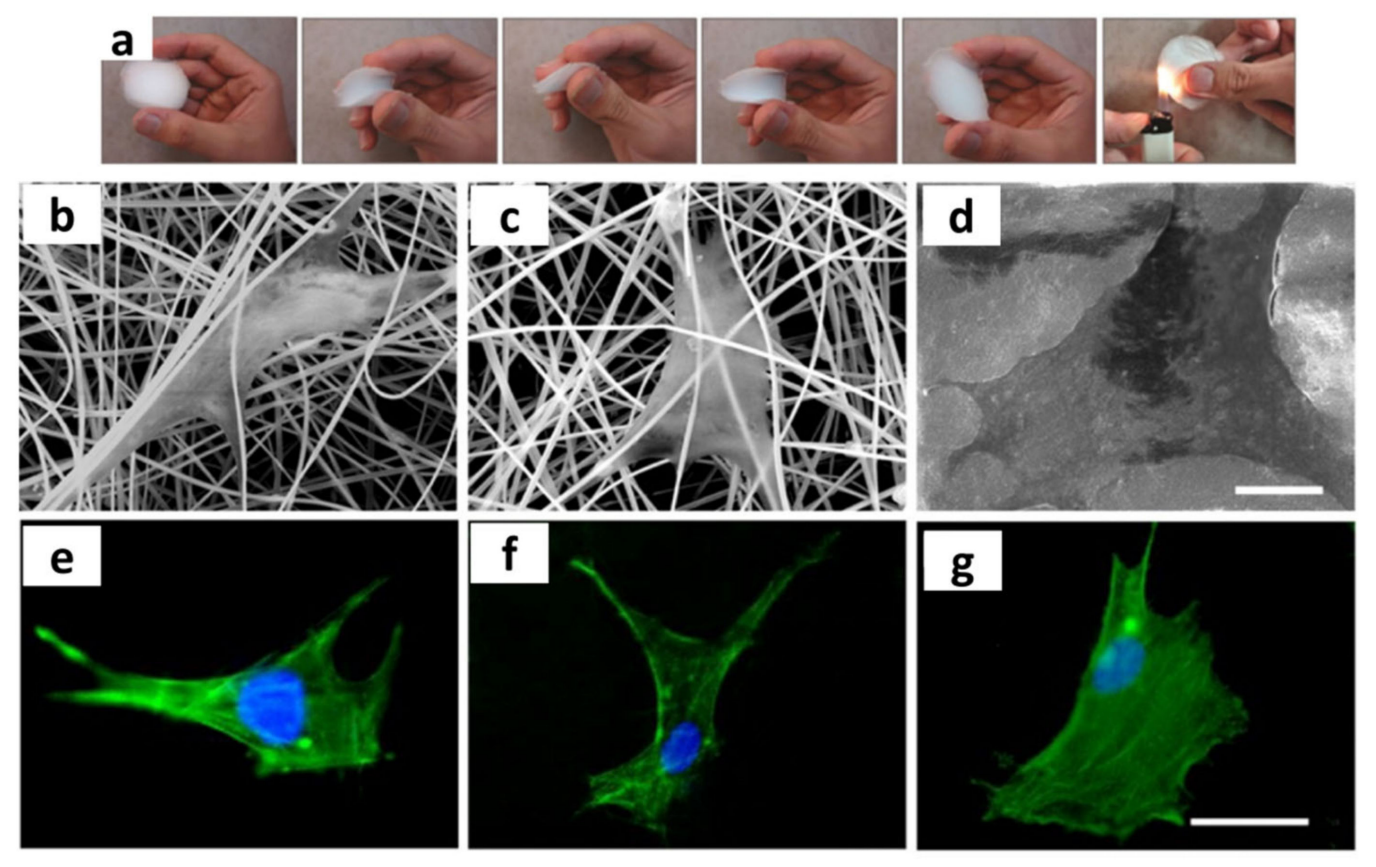
(**a**) Flexibility of YSZ nanofiber mats. Characterisation of the cell morphology and actin cytoskeleton organisation of hMSCs after 3 d of culturing in BM. Representative SEM images of hMSCs on (**b**) microwave-annealed (MW 5 °C/min) nanofiber scaffolds, (**c**) conventional oven-annealed (CO 5 °C/min) nanofiber scaffolds and (**d**) YSZ bulk disk scaffolds. Representative images of actin cytoskeleton organisation of hMSCs on (**e**) microwave-annealed (MW 5 °C/min) scaffolds, (**f**) conventional oven-annealed (CO 5 °C/min) scaffolds and (**g**) YSZ bulk disk scaffolds (actin filaments labelled with phalloidin are shown in green, and nuclei labelled with DAPI are shown in blue). Images used from Gazquez et al. [97], reprinted with permission from ACS, copyright© 2016.

Electrospinning is a unique method in which an electric field induced by a high direct-current voltage between a roller connection and a needle tip is used to spin bioceramic fibers with a constant diameter into an interconnected flexible bioceramic scaffold [94,95,96]. With the increasing demand for scaffolds with nano-to-microscale simulation of the configurations of the extracellular matrix (ECM), in 2016, Gazquuez et al., proposed the fabrication of YSZ nanofiber oriented scaffolds for bone-tissue restoration via electrospinning a mixture of YSZ and polyvinylpyrrolidone [97]. In contrast to rigid bone ceramics, the designed YSZ scaffolds have excellent flexibility at the macroscale (Figure 13a). In an in vitro bioactivity survival test using hMSCs outputs, the nanofibrous YSZ scaffolds supported the attachment and proliferation of the seeded mesenchymal stromal cells, as confirmed by SEM and hMSC phalloidin staining analyses (Figure 13b–g). The authors proposed an alternative approach for designing flexible zirconia scaffolds to traditional fragile ceramics for bone-tissue engineering. In a recent study, Thakare et al., focused on the engineering of PCL/zirconia composite nanofiber scaffolds by varying the amount of zirconia from 6% to 30% via the electrospinning process [98].

The interwoven PCL/zirconia composite nanofiber scaffolds fabricated by the author had high material stability and porosity, which are favourable for imitating the dynamic atmosphere of natural tissues. Compared with the pristine PCL scaffold, PCL/zirconia composite nanofiber scaffolds exhibited controlled degradation, swelling and superior bioactivity. Additionally, the PCL/zirconia composite nanofiber scaffolds were nontoxic, as indicated by a cell-viability test using the 3T3 mouse fibroblast cell line. This study further confirmed that electrospinning can be used to fabricate PCL/zirconia composite nanofiber scaffolds that are favourable for tissue-engineering.

Additionally, Esfahani et al., conducted electrospinning of polyamide 6(PA6)/HA on the surface of the ZTA nanocomposites with improved bioactivity suitable for bone-repair applications [99]. Even though this study did not involve electrospun scaffolds, the embedding of 6(PA6)/HA is an innovative strategy that can be used in future research involving the fabrication of electrospun 6(PA6)/HA/zirconia scaffolds. Later, various researchers investigated different precursors and design strategies for fabricating flexible zirconia scaffolds via electrospinning [100,101,102]. Even though they successfully spun zirconia scaffolds and claimed that they are potentially suitable for bone-tissue engineering, there is a lack of in vitro and in vivo studies supporting these claims.

## 4. Hybrid Techniques

In contrast to the foregoing replica techniques, Feng et al., designed an acrylonitrile butadiene styrene (ABS) template with organised channel openings using a 3D Rapid Prototyper to construct zirconia scaffolds with different shapes (cubic and cylindrical) and porosities [103]. The authors coated the zirconia scaffolds with mesoporous bioglass (MBG) to enhance their bioactivity. For the cubic and cylindrical samples, the MBG coating increased the compressive strength from 34.22 to 55.01 MPa and from 44.35 to 123.32 MPa, respectively. The cell proliferation of BMSCs on coated and uncoated samples indicated better biocompatibility.

Sapkal et al., developed a biocomposite scaffold by infusing 10%, 20%, 30%, 40% and 50% zirconia with a β-TCP background via an indirect casting technique. The ABS was first printed using a conventional 3D printer, followed by the impregnation of a zirconia/β-TCP ceramic slurry and subsequent annealing to obtain a zirconia/β-TCP bioceramic scaffold. Osteosarcoma cell line (MG-63) results and compressive-testing results for a sample with a zirconia content of 30% indicated that it was suitable for bone-tissue engineering. Scaffold fabrication with proper porosities suitable for cortical and cancellous bone must be verified [104].

**Figure 14:**
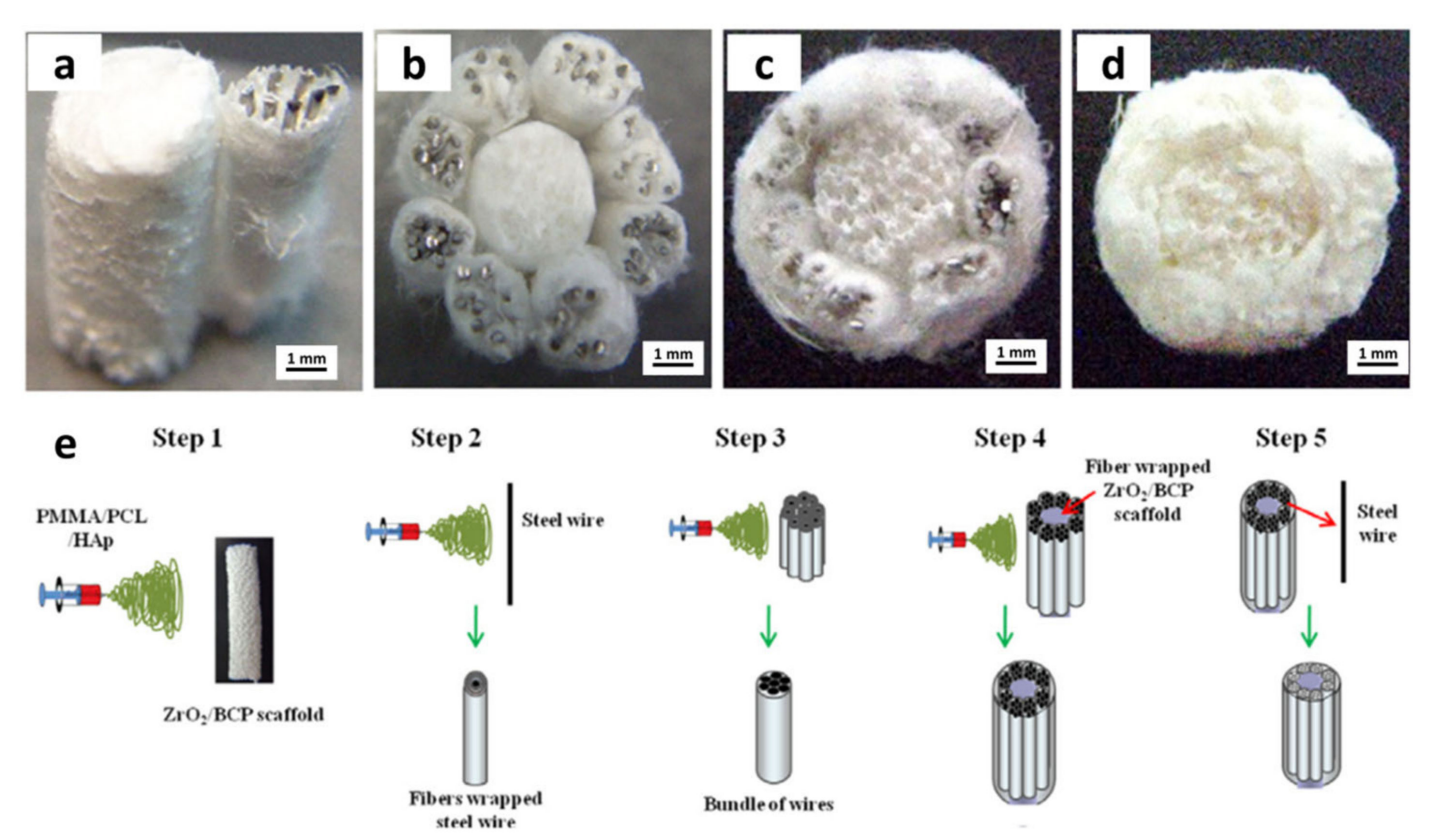
Photographs (**a**–**d**) and schematic (**e**) of the production of artificial bone. (**a**) A bundle of wires attached to the zirconia/BCPscaffold; (**b**) zirconia/BCP scaffold surrounded by bundles; (**c**) bundles surrounded by PCL/PMMA/HA; (**d**) after the steel wires were removed. Reprinted with permission from Lee et al. [105]**,** copyright© 2011.

In the aforementioned studies, the direct fabrication of flexible zirconia-containing composite scaffolds via electrospinning was demonstrated. However, a pioneering methodology combining the traditional sponge replica and modern electrospinning methods for fabricating an artificial small bone was reported by Lee et al., in 2011 [105]. Zirconia-BCP/polymethylmethacrylate-PCL-HA (zirconia-BCP/PMMA-PCL-HA) was engineered by combining the sponge replica and electrospinning techniques. First, to mimic the cancellous bone, the authors fabricated a zirconia/BCP scaffold with an interconnected porous structure via the conventional sponge replica technique (zirconia and BCP were prepared separately for comparison). To mimic the Haversian canal zone of the bone, PMMA-PCL-HA nanofibers were fenced around a steel wire with a diameter of 0.3 mm. Finally, the osteon-like flexible fibers were collected and wrapped onto the zirconia/BCP scaffold and again electrospun with PMMA-PCL-HA fibers to produce the desired scaffold (Figure 14). The outstanding cell sustainability properties (MG-63 osteoblast-like cells) confirmed the cytocompatibility of the intentionally fabricated artificial bone (zirconia-BCP/PMMA-PCL-HA scaffold). The synthetic bone-like scaffold proposed by Kim et al., that aims to replicate finger and toe bones represents a significant pioneering achievement but requires further in vivo testing before clinical implementation.

**Figure 15:**
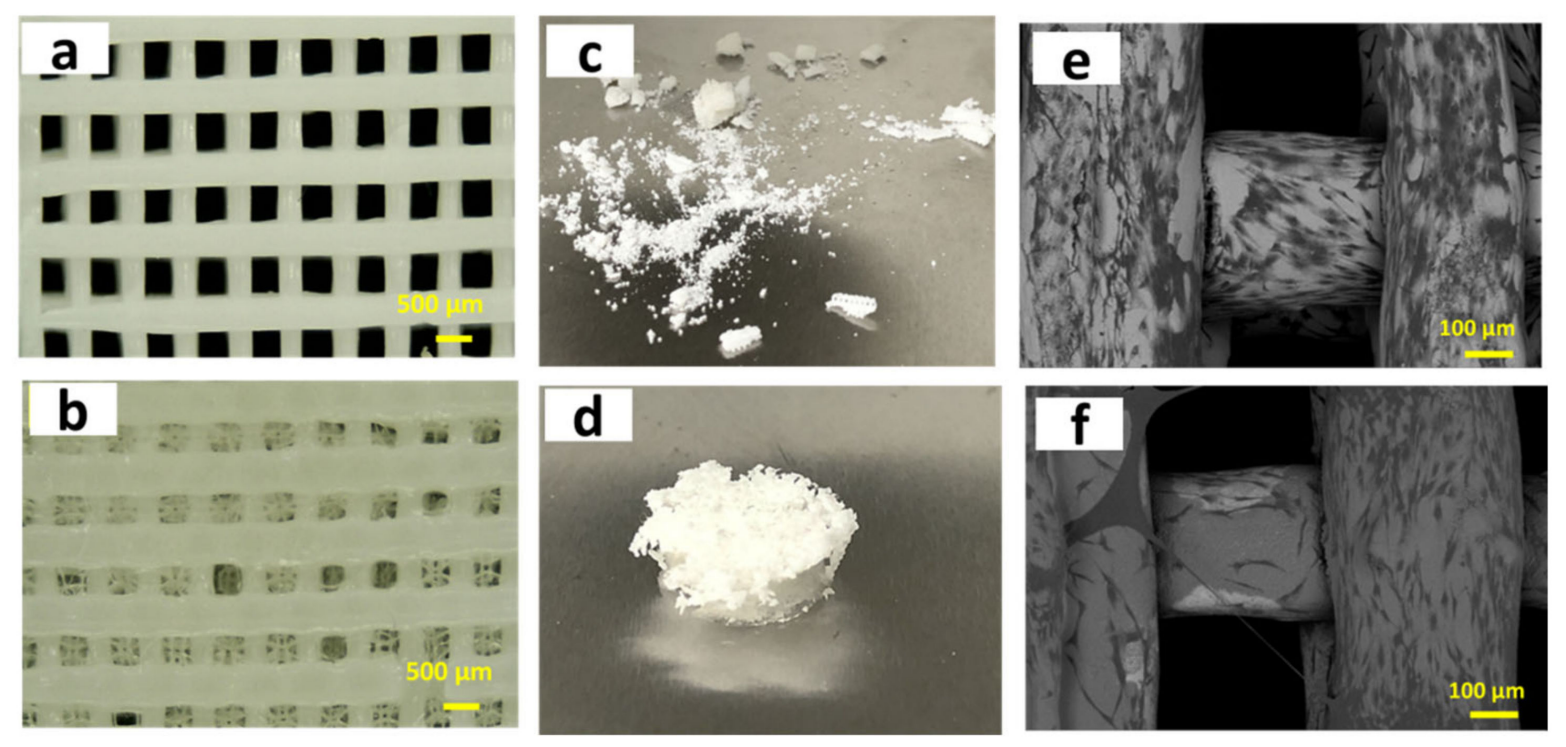
Microscopic images of (**a**) ZC and (**b**) ZCB. Digital photograph of (**c**) ZC and (**d**) ZCB after the compression test. Cell-attachment analysis using FE-SEM of the (**e**) Z and (**f**) ZC groups. Images used from Sakthiabirami et al. [106] reprinted with permission from Elsevier, copyright© 2021.

Recently, Sakthiabirami et al., fabricated porous zirconia 3D scaffolds via FDM [106]. The scaffolds were subjected to composite coating (Zn-HA/glass) on glass-impregnated zirconia to endorse hybrid roles with good mechanical and biological properties. Furthermore, the constructed scaffold was embedded with a biopolymer (alginate/gelatin) to obtain the ECM of the bone prototype (Figure 15a). The compression strength of the composite-coated scaffold and the biopolymer embedded on the composite-coated scaffold was approximately 20% higher than that of the pristine zirconia scaffold. Interestingly, the biopolymer-surrounded zirconia scaffolds were able to retain their shape, whereas the zirconia scaffolds without the biopolymer shattered into pieces (Figure 15b). This significantly affected the toughness of the biopolymer-embedded scaffold (Figure 15c). In vitro studies of hybrid scaffolds using dental pulp cells (DPCs) revealed enriched cell adhesion and cell differentiation. The authors suggested that their dynamic hybrid approach is suitable for load-bearing bone restorations; however, this should be confirmed via in vivo tests.

A summary of the zirconia-based scaffolds discussed in the previous sections that have been used for bone-regeneration applications over the past two decades is presented in Table 1. This table indicates not only the various types of scaffolds examined but also the composites/coatings used for the surface modifications. Since the bioactivity of zirconia-based scaffolds determines their clinical success, this review focused on the in vitro and in vivo outputs, along with the mechanical properties for load-bearing applications.

Very recently, Weng et.al summarized the properties and construction of ZrO_2_ and its composite materials [107]. Moreover, we have observed substantial development that has been lately accomplished in this theme. However, a review article summarizing the development trend of zirconia-based scaffolds with respect to the evolution of 3D fabrication techniques is not documented yet so far. Thus, this review presents a complete summary of the designs and strategies of 3D fabrication techniques towards highly efficient 3D zirconia-based scaffolds centered on biological (in vivo/in vitro) and mechanical developments.

## 5. Prospects and Challenges

The non-degradability of zirconia-based scaffolds is a major bottleneck of zirconia-based in comparison with other calcium phosphate bioceramics-based bone-tissue engineering applications. Considerable research attention has been directed toward resolving this concern, and zirconia-based scaffolds have seen advancements over the decades owing to the repeated efforts of bone-tissue engineers. The successful long-term use of zirconia-based implants in dental applications has been validated via clinical trials with 90% survival rates. Even though zirconia-based ceramics are found to undergo fracture in the earlier stage, the absence of surplus parasitic reactions indicates that zirconia can cause no harm to the surrounding biological environments. Hence, zirconia-based scaffolds with 3D porous architectures can provide sufficient osteointegration; once, the bone-remodelling initiated earlier fracture will not be a major issue.

**Scheme 4:**
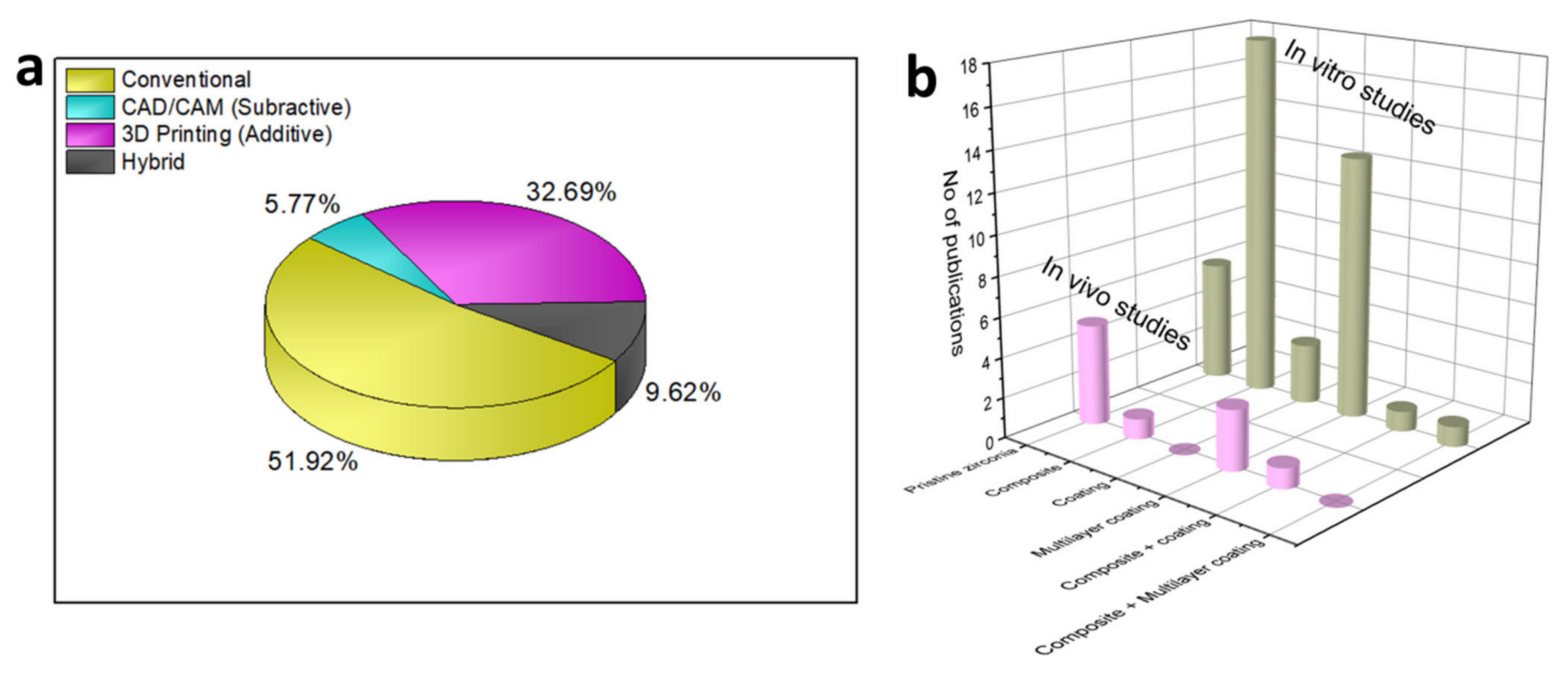
(**a**) Assessment of the recent research articles on zirconia-based scaffolds based on the fabrication techniques, (**b**) comparison of the recent research articles on zirconia-based scaffolds based on in vivo and in vitro studies.

In this paper, we comprehensively reviewed the evolution of 3D zirconia-based scaffolds over the years, detailing different fabrication techniques, e.g., the conventional foam replica technique and modern 3D-printing methods. As indicated by the pie chart in Scheme 4a, the majority of the zirconia-based scaffolds were fabricated using the conventional sponge replication technique owing to its simplicity and rapidity. This cost-effective method produces the scaffolds with sufficient porosity and mechanical stability. However, the ability of the conventional sponge replication technique to construct scaffolds with the required porosity and a precise size and shape, which is critical for defining cell migration and tissue growth, is limited. Following the sponge replication technique, numerous conventional methods, including salt leaching, freeze-drying, foam replicas, solvent casting and particulate leaching, have been proposed for designing zirconia-based scaffolds.

In the design aspects, pursuing the finest scaffold design for bone-repair applications has greatly increased; the recent evolution of 3D-printing techniques for designing 3D bioceramic scaffolds has encouraged bone-tissue engineers to construct 3D zirconia-based scaffolds with precise dimensions and interconnected porosity. Even though 3D printing is well established for calcium-based bioceramics aimed for bone-tissue engineering, few studies have been performed on 3D-printing techniques, including extrusion-based, DIW, DLP and SLS techniques, for fabricating zirconia-based scaffolds for bone-tissue engineering. This clearly indicates that 3D-printing techniques are still in a stage of infancy. Among the few studies reported thus far, a multi-extrusion technique-derived 3D zirconia-based scaffold exhibited considerable promise, with improved mechanical and biological properties. However, the surfactants, organic solvents and binders used for the 3D-printing process must be carefully selected, as they can be harmful to host cells and tissues. Given the limited number of reports on zirconia-based scaffolds fabricated via 3D-printing techniques, it may be challenging for future bone-tissue researchers to clearly understand the degradation, mechanical stability and bone-regeneration ability of 3D-printed zirconia scaffolds in load-bearing applications in human biological environments.

To address the poor interfacial compatibility between the zirconia scaffolds and incoming cells, three main strategies have been employed by bone-tissue engineers: surface reformation, interface-phase introduction and crystal-structure variation [108]. For instance, in the early 2000s, Lee et al., made a significant contribution to enhancing zirconia scaffolds for bone-regeneration applications by optimising diverse coating and fabrication techniques. The dual apatite FA/HA coating approach for nullifying the direct chemical transformation between the HA and zirconia scaffolds has been a pioneering outlook considered for future research. Additionally, bone-tissue researchers have included bioactive materials in zirconia scaffolds (resulting in composite scaffolds) for improving the bioactivity. Even though bioactive coating/composite formation increases the interfacial bonding strength, there are unresolved challenges to be addressed in future studies. For example, the inclusion of secondary phases in the zirconia scaffolds induces the chemical transformation of zirconia into undesired parasitic phases, which can reduce the strength [109]. Additionally, the mechanical and physical impacts of secondary bioactive coatings must be considered before practicing in bone defect locations.

Finally, it is essential to discuss the clinical possibilities of zirconia-based scaffolds. The direct comparison of the strength of the zirconia-based scaffolds to the strength of the natural bone (cortical bone) lacks among the reviewed articles, which is very essential to understand the ability of zirconia-based scaffolds to satisfy the critical bone defects requirements. In most of the collected literature, the in vitro studies focused on validating the metabolic activity of osteogenic cells (Scheme 4b). Surprisingly, there is a lack of in vivo proof of zirconia-based scaffolds in animal biological environments. Previous reports suggested that there is a large inconsistency between the in vitro performance and the in vivo performance [110]. In most of the reviewed literature on zirconia-based scaffolds, little attention was paid to the in vivo evolutions. In particular, zirconia-based scaffolds formulated via polymeric replication have been subjected to in vitro and in vivo evolutions. Indeed, polymeric replication-formulated zirconia-based scaffolds have been demonstrated to replicate hard-cortical and spongy-cancellous bone.

Few 3D scaffolding technologies have been commercially available, among which CAD/CAM is commonly used and the most dependable. After the successful fabrication of zirconia-based scaffolds for dental applications using the CAD/CAM technique. Apart from the in vitro and in vivo performance, 3D zirconia-based scaffolds fabricated using the CAD/CAM technique has been successfully employed for human mandibular reconstruction. Even though 3D-printed zirconia-based scaffolds were claimed to be the best possible alternatives to the conventional technologies, few in vivo studies support the clinical possibilities of 3D-printed zirconia-based scaffolds. Hence, it is essential to closely monitor the in-depth reproduction of natural mechanical and biological inducements and the rate of vascularisation of 3D-printed zirconia-based scaffolds in the in vivo environment. Multifaceted research should be targeted to systematically appreciate the perspective of 3D printing in bone-restoration applications.

## 6. Conclusions

The primary goal of the scaffold is to get completely absorbed and replaced by body tissue. However, in large defects, scaffold or implanted artificial bone materials like HA or TCP are not absorbed completely and remained for a long time in vivo studies. And the healing period of each bone is different from several weeks to several months (ex. Femur 6 months). Until now researchers are not able to control the absorption rate of scaffold depending on new bone formation. Over the past 20 years, the zirconia-based scaffold has played a substantial role in critical-sized bone-defect applications, following the tremendous success of modern restorative dentistry. For the large sized loading area, a zirconia-based scaffold that has comparable strength to that of natural bone is needed to support bone formation during the long healing time. However, it remains challenging for orthopaedic experts and bone-tissue researchers to heal critical-sized bone deficiencies using zirconia-based scaffolds, owing to the immature fabrication techniques and unfavourable composite/coating materials for enhancing the bioactivity. Clinically feasible possibilities for promoting surface activation, along with appropriate 3D fabrication practices, are likely to be a focus area for future zirconia-based -scaffold design. The advancements in material engineering in association with biomechanical engineering could be critical in determining zirconia-based scaffolds as a successful biomaterial. Undoubtedly, with the expansion of advanced 3D-printing technology and unique coating strategies, the liability of zirconia-based scaffolds for critical-sized bone-replacement applications is not far away.

## Figures and Tables

**Table 1:** A summary of the zirconia based scaffolds for bone tissue engineering applications based on fabrication/design and its properties.

Year	Materials	Ceramic Content	Fabrication Techniques	Composite Materials	Infiltration or Intermediate Layer	Coating Materials	Porosity (%)	Pore Size(µm)	Mechanical Properties			Biological Properties		Ref.
									CS (MPa)	Modulus	Others	In Vitro	In Vivo (weeks)	
Conventional Technique														
2003	YSZ-FA-HA	–	Sponge replica	–	FA ( ~ 5µm)	HA ( ~ 20µm)	74–92	~ 600	1.6–35	-	Adhesion strength ~22MPa	HOS cells	–	[42]
2004	YSZ-Ca P	–	Sponge replica	–	FA	Ca P (HA,FA,TCP,HA+FA,HA+TCP)	~ 90	500–700	–	–	–	SBF, MG-63 and HOS cells	–	[43]
2004	YSZ (5.2 wt% Y)-FA-HA	–	Sponge replica	–	FA	HA	90	~ 600	–	–	Adhesion strength ~33MPa	Saline and MG-63 cells	–	[44]
2008	YSZ-FA-HA	–	Sponge replica	–	FA	HA	~ 84–87	~ 500–700 and ~ 150–200	~ 7-8	–	–	–	Rabbit calvarial defect (4,12)	[45]
2010	YSZ—MgO doped alumina	60 wt% ( ~ 20 vol%)	Pore former (Expanded polystyrene beads) and vacuum slip casting	YSZ (5, 10, 20 vol%)	–	58S33C or 58S bioglass (15–20µm)	–	262–121	7.52–5.42	1.64–1.17 GPa	–	SBF	–	[63]
2011	YSZ-BCP	YSZ (10 vol%)	Sponge replica	–	BCP/YSZ (1:1)	BCP (top layer)	68.3	100–250	7.2	–	–	MG-63 cells	–	[50]
2012	YSZ-HA	–	Sponge replica	HA (20, 30, 40, 50 wt%)	-	Fibrin gel (with BMSCs encapsulation)	72–91	–	2.5–13.8	–	–	MCT3-E1 and BMSC cells	Rat calvarial defect (3,6)	[20]
2013	YSZ-bioglass-HA	–	Freeze drying	HA (18%)	45S5 bioglass	HA ( ~ 20µm)	68.2	100-500	2.11	–	Flexural strength 343 MPa; Vickers hardness (substrate) 5.22 GPa	–	–	[59]
2013	YSZ-HA	–	Pore former (PMMA beads and starch powder)	HA (30 vol%)	1st layer-YSZ (20 and 10 vol%) and 2nd layer-HA+YSZ(5 vol.%) +Al_2_O_3_ (5 vol.%)	HA (pure)	–	1–10 and 20–50	–	–	Bending strength 320–390 Mpa and Adhesion strength 24.5MPa	L929 cells	–	[111]
2014	YSZ-Ca P	–	Sponge replica	β-TCP (50, 60, 70 wt%)	–	–	65–84	–	4.95–6.25	48–63 MPa	–	h-ESC cells	–	[48]
2014	YSZ- β-TCP-HA	–	Sponge replica	–	–	β-TCP, HA and BCP	–	200–500	–	–	–	MC3T3-E1 cells	–	[51]
2015	YSZ-BCP-Collagen	–	Sponge replica	–	BCP/YSZ (1:1) ( ~ 10–15µm)	BCP (top layer) ( ~ 10–15µm) and collagen	–	150–500	6.8	–	–	MC3T3-E1 cells	Rabbit femoral defect (1 and 5 months)	[52]
2015	YSZ	–	Sponge replica	–	–	–	92.7–68	830–577	0.6–4.4	–	–	SBF and BMSC cells	–	[57]
2015	YSZ -Al_2_O_3_	–	Pore former (polyethylene beads)	Al_2_O_3_ (20wt%)	–	–	50–61	113–185	60–240	3–10 GPA	–	MC3T3-E1 cells	–	[112]
2015	YSZ and MgSZ	–	Pore former (polyethylene beads)	Y_2_O_3_ (8, 3 wt%) and MgO (8, 3 wt%)	–	–	50 and 57	167 and 144	210 and 120	10 and 6	–	MC3T3-E1 cells	–	[64]
2015	ZrO_2_-CS-SF	–	Freeze drying	ZrO_2_ (1)-CS (2)-SF(2.5)wt%	–	–	>75	50–150	–	–	–	PBS and HGF cells	–	[61]
2015	ZrO_2_- POM-SF	–	Freeze drying	POM-ZrO_2_-SF	–	–	–	100–200	–	–	–	SBF and HGF cells	–	[60]
2017	YSZ-HA-PLA	–	Solvent casting and salt-leaching	PLA-HA (10, 15, 20 wt%)-YSZ (10, 15, 20 wt%)	–	–	–	–	0.8–6.9	0.7–5.1 MPa	Flexural strength 0.09–0.47 MPa; Flexural strain 17.5–8.2%	SBF	–	[66]
2018	YSZ	–	Sponge replica(crushed into granules after sintering)	–	–	–	–	300–400	–	–	–	–	Rabbit calvarial defect (2, 4, 8)	[27]
2018	YSZ-FA-HA	55	Sponge replica	–	FA ( ~ 45.7µm)	HA-PRP/HS	71.6	310 ± 150	2.98–18.8	–	–	MG-63cells	–	[46]
2018	ZrO_2_- HA-PRP/HS	–	Sponge replica	–	FA	HA and Loaded with PRP gel/HS	–	–	–	–	–	–	Rabbit mandible defect (6,8)	[47]
2018	ZrO_2_	–	Solvent casting and Salt-leaching	–	–	–	79	100	–	–	–	–	Case study (repair of oroantral fistula)	[67]
2018	ZrO_2_-CS-HA -CZ	–	Freeze drying	CS(2.5wt%)-HA- ZrO_2_-CZ	–	–	85–94	10–100	0.3–1	CM-0.24–0.97 MPa;EM- 3–4 GPa	–	PBS and OB-6 cells	–	[62]
2019	YSZ	50–70 wt%	Sponge replica	–	–	58S bioglass ( ~ 2µm)	94–85	700–322	–	–	–	MG-63cells	–	[55]
2020	YSZ	60wt%	Sponge replica	–	–	–	94–85	700–120	0.2–0.8	9–39 MPa	FEA, hardness (387 MPa)	–	–	[58]
2020	YSZ-58S	60 wt%	Sponge replica	–	58S bioglass (infiltration)	58S bioglass	93–89	–	0.33–0.44	–	–	SBF and MG-63 cells	–	[56]
2020	YSZ-HA-TCP	55 wt%	Sponge replica	YSZ (5, 10, 15, 20 and 25 wt%)	–	–	65.7–84.4	–	13.2–4.5	–	–	SBF	–	[49]
Extrusion Based Printing														
2011	YSZ—HA(α –TCP)	48–43 vol%	Multipass extrusion	–	HA (α-TCP)—YSZ	HA	77	86	53	–	–	MG-63 cells	–	[79]
2012	YSZ—HA	45–40 vol%	Multipass extrusion	–	HA—YSZ	HA	–	–	7–20	–	–	MG-63 cells	–	[77]
2012	YSZ—BCP	46–41 vol%	Multipass extrusion	–	YSZ—BCP	PCL/BCP	92–78	–	8.27–12.7	–	–	MG-63 cells	–	[78]
2014	YSZ	70 wt%	Direct ink writing (DIW)	–	–	–	55 and 63	–	8 and 10	–	–	HCT116 cells	–	[23]
2017	ZrO_2_—β-TCP	–	3D Bio-plotter	ZrO_2_ (30wt%)	–	–	60–76.46	160–226	7–12.025	–	–	MG-63 cells	–	[79]
2018	YSZ-Al_2_O_3_(ZTA)	70wt% (35.5 vol%)	Robocasting	ZTA (YSZ-16 wt.%)	–	–	50	245	–	–	–	HOB cells	–	[87]
2018	ZrO_2_-BCP	–	FDM	ZrO_2_ (10 wt%)	–	–	–	350	0.5	–	–	MG-63 and hMSCs cells	–	[81]
2019	YSZ	48 vol%	Robocasting	–	–	–	–	200–500	–	–	–	–	–	[85]
2019	ZrO_2_—β-Ca_2_SiO_4_	–	3D Bio-plotter	ZrO_2_ (5, 10, 15 wt%)	–	–	~ 67	–	3.9–6.1	–	–	SBF and BMSC cells	Rat calvarial defect (8)	[80]
2020	ZrO_2_—PCL	–	FDM	ZrO_2_ (5, 10, 20 wt%)	–	–	46.2–47	459.2–462.7	5.5–7.9	43–67 MPa	–	MC3T3-E1 cells	–	[83]
2021	YSZ-FA-HA	39.5 vol%	Direct ink writing (DIW)	–	FA	HA ( ~ 20µm)	61.1–75.3	–	20.8–62.9	–	–	SBF	–	[86]
Polymerization Based Printing Technique														
2019	ZrO_2_ -HA	60 wt%	DLP	ZrO_2_ (1, 3, 6 wt%)	–	–	–	–	–	–	Tensile strength (29.4%); Bending strength (23.9%)	BMSC cells	–	[26]
2020	YSZ-HA	–	DLP	HA (10, 20, 30 wt%)	–	–	54.6	–	52.25	–	CS after soaking in SBF (25 MPa)	SBF and MC3T3-E1 cells	–	[91]
CAD/CAM Technique														
2019	YSZ	–	CAD/CAM (5-axis milling)	–	–	–	45.06	–	52.25	–	FEA	SBF	–	[69]
2019	YSZ	–	CAD/CAM	–	–	–	–	–	–	–	–	–	Case study (mandibu-lar defects)	[68]
2021	YSZ	–	CAD/CAM	–	–	–	–	–	–	–	–	MC3T3-E1 cells	Rat femur (2, 4, 8)	[70]
Electrospinning														
2016	YSZ- PVP	–	Electrospinning	–	–	–	–	–	–	1.11MPa	–	HMSCcells	–	[97]
2017	ZrO_2_-PCL	6–30 wt.%	Electrospinning	–	–	–	–	–	–	–	–	3T3 cells	–	[98]
SLS Technique														
2014	ZrO_2_-CaSiO_3_	–	SLS	ZrO_2_ (10, 20, 20, 40 wt%)	–	–	70	1600	17.9–44.1	–	Fracture toughness 1.14–1.66 MPa.m^1/2^	SBF and MG-63 cells	–	[93]
Hybrid Technique														
2011	YSZ-TCP	–	3D Rapid Prototyper (ABS template)followed by slurry impergation	–	–	Mesoporous bioglass	63–68	500–800	44.35–123.32	–	–	SBF and BMSC cells	–	[103]
2011	YSZ-BCP and PMMA-PCL-HA (Fiber)	10 vol%	Sponge replicaAnd Electrospinning	–	YSZ-BCP	BCP	67.68–69.65	–	4.83–4.97	–	–	MG-63 cells	–	[105]
2012	ZrO_2_	50 vol.%	Free-form	–	–	–	40	350	–	–	–	–	Case study (maxilla)	[113]
2016	ZrO_2_—β-TCP	–	3D Rapid Prototyper (ABS template) followed by impergation	ZrO_2_ (10, 20, 30, 40, 50 wt%)	–	–	68.5–82.5	–	3–15	184–396 MPa	–	PBS and MG-63 cells	–	[104]
2020	YSZ-bioglass-Zn-HA-Biopolymer	40 Vol%	FDM and Freeze drying	–	Glass (Infiltration)	Glass/Zn-HA(~ 1µm) and Gelatin/alginate	~ 40%	300–450	68.2–89.8	1.7–2.6 GPa	Strain energy density 1.8-4.2 MJ/m^3^	DPCs cells	–	[106]

## Data Availability

All the data were collected with copyrights permission.

## Abbreviations

YSZ—3mol.% yttria stabilised zirconia, FA—Fluroappatite, HA—Hydroxyapatite, CaP—Calcium phosphate, TCP—Tricalcium phosphate, MgO—Magnesium oxide, BCP—Bicalcium phosphate, PMMA—Polymethamethylacralate, β-TCP-Beta—Tricalcium phosphate, Al2O3—Aluminium oxide/Alumina, MgSZ—Mgnesium stabilized zirconia, Y_2_O_3_—Yttrium oxide, ZrO_2_—Zirconium dioxide/Zirconia, CS—Chitosan, SF—Silk fibrin, POM—Polyoxometalates, PLA—Polylatic acid, PRP—Plasma rich protein, HS—Heparin sulfate, CZ—Calcium zirconate, α-TCP-Apha—Tricalcium phosphate, ZTA—Alumina toughened zirconia, Ca_2_SiO_4_—Calcium silicate, PCL—Polycaprolactone, PVP—Polyvinylpyrrolidone, ABS—Acrylonitrile butadiene styrene, Zn-HA—Zinc doped hydroxyapatite, DIW—Direct ink writing, FDM—Fused deposition modelling, DLP—Digital light processing, CAD/CAM—Computer aided design/Computer aided milling, SLS—Selective laser sintering, CS—Compression strength, HOS—Human osteoscarcoma, SBF—Stimulated body fluid, MG63—Ostesarcoma cells, MCT3-E1—Murine preosteoblast cells, BMSC-Bone marrow—derived mesenchymal stem cells, L929—Murine fibroblast cells, PBS—Phosphate buffered saline, HGF—Human gingival fibroblast cells, OB6—Murine bone marrow-derived osteoblastic cells, HCT116—Human colon carcinoma cells, HOB—Human osteoblast cells, hMSC—Human mesenchymal stem cells, DPCs—Dental pulp cells.
